# On the multiple solutions of the complex absorbing potential method for resonances

**DOI:** 10.1039/d6sc03887e

**Published:** 2026-09-04

**Authors:** Fábris Kossoski

**Affiliations:** a Univ Toulouse, CNRS, LCPQ Toulouse France fkossoski@irsamc.ups-tlse.fr

## Abstract

The complex absorbing potential (CAP) method is an effective approach to model anionic resonances. However, the existing optimization criteria for the CAP strength give rise to multiple solutions, and the common practice has been to pick a single one as describing the resonant state and to discard the rest as unphysical. Here, we challenge this view by demonstrating that several solutions can be acceptable approximations for the resonance. This ambiguity introduces an important source of uncertainty in CAP calculations that we show to be generally larger than the errors stemming from the basis set incompleteness and the approximate electronic structure method, affecting energies and properties alike. Moreover, various strategies that have been proposed to mitigate the appearance of multiple solutions are shown to be ineffective, like enlarging the basis set, subtracting the energy contribution from the CAP, and introducing a real perturbative potential. Although the CAP method has proven merits in the description of anionic resonances, the existence of multiple solutions emerges as a critical and unsolved problem.

## Abstract 2

Calculations using the complex absorbing potential method yield multiple valid solutions for anionic resonances. This lack of uniqueness cannot be mitigated by current approaches and represents a significant source of uncertainty.

## Graphical abstract

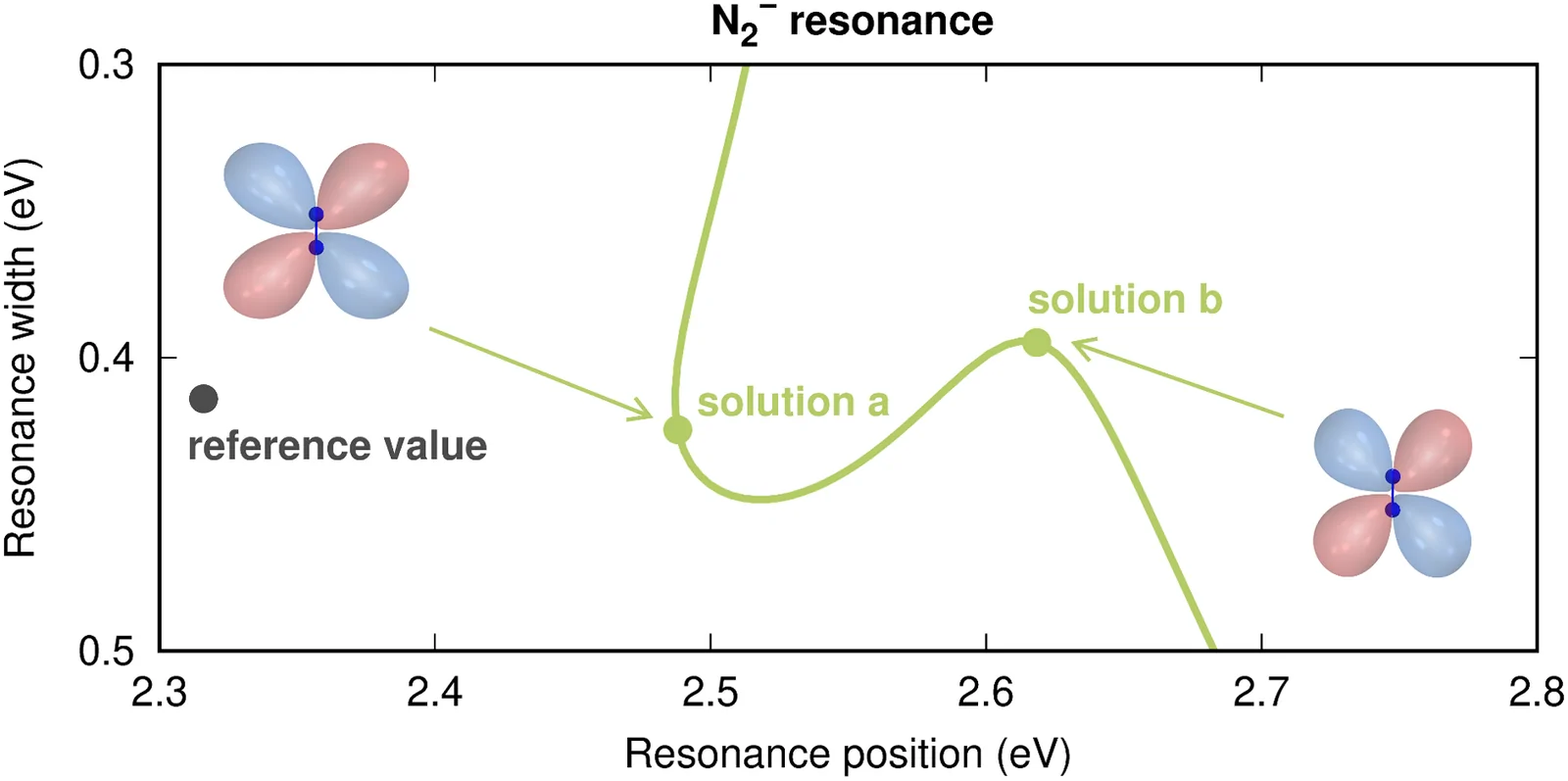

## 1 Introduction

Molecular systems have bound and unbound electronic states. Embedded in the unbound part of the spectrum, one finds resonances. These are transient states that survive for long enough to leave observable signatures, yet not enough to be mistaken by a bound state, which is stable. Anionic resonances^1–4^ and core-vacant states^5–7^ are examples of resonances in molecular systems.

In contrast to electronically bound states, resonances can decay by ejecting one electron. Because of its finite lifetime, a resonance is characterized by a complex-valued energy,

1 *E* = *E*_R_ − *iΓ*/2

*E*_R_ is the resonance position, which places the resonance with respect to the electron detachment threshold, and *Γ* is the resonance width, whose inverse is the lifetime against electron detachment.

To model resonances, one needs to describe the many-body electronic structure problem while treating the continuum of free electron states. Among the various theoretical approaches that propose to do so,^8–20^ the complex absorbing potential (CAP) method^16^ has established itself as a competitive approach, particularly for describing anionic resonances.

Before describing the CAP method itself, it is worth mentioning the continued effort of combining it with a wide range of electronic structure methods. So far, it has been integrated with multireference configuration interaction (MRCI),^21,22^ second- and third-order algebraic diagrammatic construction [ADC(2) and ADC(3)],^23–25^ Fock-space multireference coupled-cluster,^26,27^ symmetry-adapted-cluster configuration interaction (SAC-CI),^28^ density functional theory,^29,30^ equation-of-motion electron-attachment coupled-cluster with singles and doubles (EOM-EA-CCSD),^31,32^ extended multiconfigurational quasidegenerate second-order perturbation theory,^33^ extended multistate complete active space second-order perturbation theory (XMS-CASPT2),^34^ electron propagator based on multiconfigurational self-consistent field,^35^ selected configuration interaction,^36^ second-order approximate coupled-cluster,^37^ and the GW method.^38^ Some of these implementations have also been combined with the projected CAP formalism,^39^ an approximate but faster alternative to the standard CAP methodology.

Beyond single-point energy calculations, a series of important developments in recent years have enlarged the scope of problems that can be tackled with the CAP method. Analytical nuclear gradients at the CAP/EOM-EA-CCSD level^40,41^ have enabled efficient optimization of equilibrium geometries for resonances,^42^ the location of minimum energy crossing points between anion and neutral states,^43^ and of minimum energy exceptional points (MEEP), where two anionic resonances become degenerate.^44^ More recently, the calculation of nonadiabatic couplings have also been implemented.^41,45^ These are key steps to advance methodologies that simulate the dynamics of anionic resonances.^46–49^

Now turning our attention to the CAP method, it consists in perturbing the physical Hamiltonian *Ĥ*_0_ by an absorbing potential −*iηŴ*,^16^

2 *Ĥ*(*η*) = *Ĥ*_0_ − *iηŴ*

where *Ŵ* is a one-body potential that defines the form of the CAP while *η* defines its strength. The CAP perturbation has the purpose of absorbing the diverging and oscillatory part of the resonance wave function, thus implicitly accounting for the continuum states. In the complete basis set limit, the CAP method yields the exact resonance parameters in the limit *η* → 0.^16^

In practice, however, a finite basis set implies that the continuum is described incompletely. Taking the limit *η* → 0 would only produce a discretized set of continuum-like states, some of which having partial resonant character. As a consequence, larger values of *η* are needed to disentangle continuum and resonant states. However, as the CAP is an artificial potential, increasing *η* also disturbs the system. Therefore, one tries to find an optimal value *η*_opt_ that balances out the error associated with an implicit description of the continuum (which decreases with *η*) and the error introduced by the CAP itself (which increases with *η*). Finding this trade-off lies at the core of CAP calculations.

However, optimizing the CAP for a given resonance often yields multiple solutions for *η*_opt_. How to rationalize more than one CAP solution? What is commonly done in the field is to take a single solution as physical, and to discard all the others, labeled unphysical, spurious, or artifacts.

The main goal of this contribution is to challenge this view. To start, electing and reporting a single CAP solution as “physical” has never been justified. In fact, the CAP formalism^16^ provides no guarantee that only one solution should be expected in finite basis set calculations. Rather, when a single solution is elected, one makes an additional assumption that only one is “physical”, even though the term is hardly defined. Besides, proceeding along this line leads to serious inconsistencies, as will be discussed below. With few exceptions,^38,40,50,51^ the very existence of multiple CAP solutions is rarely mentioned. Most works report a single pair of resonance position and width for a given resonant state. One is left with two possible interpretations: either other CAP solutions do not exist or the justification for electing a single one is trivial and not worth mentioning. Here, we will show that neither of the two possibilities is generally valid.

We propose a different way to rationalize the multiple CAP solutions. We start by lifting the assumption that only one would be acceptable. Instead, we take each solution at face value: a particular way in which the combined error from the approximate continuum and the CAP perturbation is minimized. In fact, we will show that various CAP solutions display the expected features of a resonant state, while spanning a qualitatively comparable range of energies. As such, they should be seen as different approximations of the resonance. We do not claim that all CAP solutions are equally reasonable approximations, which would be the other extreme of what is usually done: picking just one as physical.

To support our discussion, we address two systems that have been extensively studied with the CAP method: the π* shape resonance of N_2_^−^,^22,32,36,38,39,49,51–54^ and the π* and σ* shape resonances of the chloroethylene anion.^34,34,43,44,55^ The resonances are described at the equilibrium geometries of the neutral molecules, at the MEEP geometry of the chloroethylene anion,^44^ and as a function of the internuclear distance of N_2_. In addition to performing new CAP calculations, we also revisit some of the previous calculations, which allow us to clarify some inaccuracies.

The remaining of the text is organized as follows. Sec. 2 details the methodology employed in this work, covering the procedure for the CAP optimization (Sec. 2.1), and the computational details of the calculations (Sec. 2.2). Our results are presented and discussed in Sec. 3, which is organized in seven subsections. The first (Sec. 3.1) raises the problem of multiple solutions in CAP calculations. Each of the following four subsections explores the role of a different aspect on the structure of multiple solutions: the electronic structure method (Sec. 3.2), the basis set (Sec. 3.3), the optimization criteria and the CAP shape (Sec. 3.4), and the addition of a real perturbation to the CAP (Sec. 3.5). The last two subsections focus on key shortcomings of potential energy surfaces obtained with the CAP method: their non-smoothness (Sec. 3.6), and their non-unique critical points (Sec. 3.7). Sec. 4 summarizes our main conclusions and perspectives.

## 2 Methodology

### 2.1 Stationarity of the velocity

First, we would like to comment on a subtle point regarding the optimization of *η*.

The most common optimization protocol, originally proposed by Riss *et al.*,^16^ is to minimize the first order term of a Taylor expansion of the energy, also called the logarithmic energy velocity,3
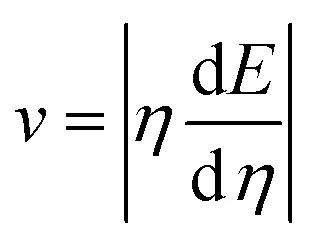
Here, *v* is referred to as velocity, for simplicity. The velocity optimization criterion is commonly stated as4
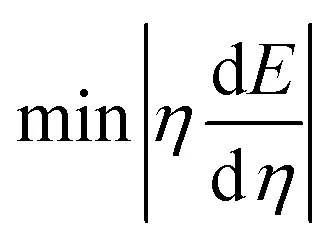


However, this expression does not represent what is done in practice. One does not search for the minimizer of the velocity, but rather for its local minima. Notice, for instance, that *η* = 0 is always the minimizer of eqn (4), even though it does not make the velocity stationary for a purely imaginary CAP.

Instead, the optimal value of *η*, *η*_opt_, is obtained where the velocity has a local minima, *i.e.*5
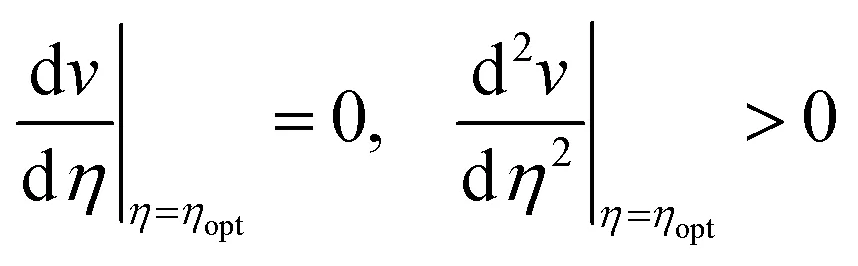


Clearly, this is not a new optimization criterion on itself. It is, however, the correct statement of what is actually done.

In practice, one solves for the CAP-augmented Hamiltonian [eqn (2)] over a range of *η*. The obtained complex-valued energies *E*(*η*) form what is called a *η*-trajectory in the complex plane. Then, a CAP solution is identified at a value of *η*_opt_ that satisfies eqn (5), which also manifests as an inflection of the *η*-trajectory. The smaller the *v*, the sharper is the inflection, and the closer to stability is the resonance according to this criterion. The resonance energy is evaluated as *E*(*η*_opt_), which is referred to as the 0th-order energy.

A common strategy to mitigate the artificial perturbation of the CAP is to remove its energy contribution to first order.^16^ This is done by defining the 1st-order (also called corrected or deperturbed) energy,6
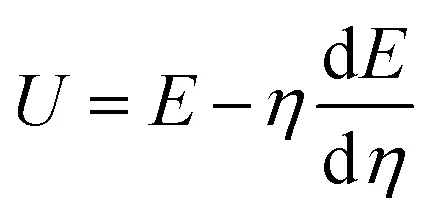
and finding stationary values of its associated velocity, eqn (3), with *E* being replaced by *U*. Here, we adopt the terms 0th- and 1st-order energies for *E* and *U*.

While our focus is the velocity criterion [eqn (5)],^16^ as it is the most widely used one, we also discuss alternative optimization procedures.^56–59^

### 2.2 Methodological details

Most of all our calculations rely on the CAP/EOM-EA-CCSD method.^31,32^ In addition, unrestricted Hartree–Fock (UHF) and coupled-cluster with singles and doubles (CCSD) calculations are also performed independently for neutral and anionic resonant states, giving rise to the state-specific CAP/ΔUHF and CAP/ΔUCCSD models. The CAP/UHF reference wave function is obtained by using as a guess the CAP-free orbitals of the neutral and occupying the lowest-lying virtual orbital of the desired symmetry. The presence of the CAP favours convergence to a solution featuring a valence-like singly-occupied orbital. When a continuum-like solution is found instead, usually at very small *η*, we use as a guess the orbitals converged at a larger *η*, which is found to be effective to track the same UHF solution. The CAP is always engaged since the Hartree–Fock step. Core orbitals are not frozen. All calculations are performed with Q-Chem.^60^ The procedure to generate *η*-trajectories from the CAP calculations is detailed in Sec. S1 of the SI.

Following the common practice in the field, we take *Ŵ* to be zero inside a volume that encompasses the molecule, and to grow quadratically in each Cartesian direction after the frontier of this volume, which is called the CAP onset. Most of our calculations employ such a box-shaped CAP, but spherical and smooth Voronoi shapes are also used.^61,62^ Even though the choice of CAP onset has long been recognized to have a major impact on the calculation of resonance parameters, only recently this aspect has started to be investigated more systematically.^59,63^ This is not addressed here.

We employ Dunning's correlation-consistent augmented basis set supplemented with a set of extra diffuse Gaussian functions, denoted by the number of functions of each angular momentum (*e.g.* 3s3p3d). The exponents of the extra set are generated by successive divisions by two, for each angular momentum and atom, starting by Dunning's most diffuse function. The additional basis sets are placed either at the geometric center of the molecule, when they are labeled (C), or at each atom, when they are labeled (A).

For the vertical calculations for N_2_^−^, we take the geometry and CAP onsets from ref. 32, also adopted in subsequent CAP calculations.^36,38,39,51,54,64^ We perform an extensive survey of ten different basis sets, including the geometric-centered aug-cc-pVXZ+3s3p3d(C) basis sets, employed in ref. 32, and the atom-centered aug-cc-pVXZ+3s3p3d(A) basis sets, employed more recently,^54^ where X = D, T, Q. Compared to these, we present new results with four larger bases, improving the valence region with the quintuple-zeta aug-cc-pV5Z+3s3p3d(C) and aug-cc-pV5Z+3s3p3d(A) basis sets, and the diffuse region, both radially, with the aug-cc-pVTZ+6s6p6d(A) basis set, and angularly, with the aug-cc-pVTZ+6s6p6d6f(A) basis set.

Our potential energy curves of N_2_^−^ are built with exactly the same procedure of ref. 64, namely, CAP/EOM-EA-CCSD/aug-cc-pVTZ+3s3p3d(C), for a fixed CAP onset. However, we adopt a finer grid of points (0.01 Å) than in the previous calculations (0.05 Å).^39,51,64^ This allow us to carefully track the potential energy curves, revealing a number of opposite conclusions to those of previous works.

To describe the chloroethylene anion, we employ the same level of theory as in ref. 43 and 44, namely, CAP/EOM-EA-CCSD/aug-cc-pVDZ+3p(A), with the even-tempered extra functions centered at the C and Cl atoms. The calculations are performed at the two geometries (and with the same CAP onsets) reported in ref. 44: the equilibrium geometry of the neutral molecule and the MEEP geometry associated with the π* and the σ* resonances.

All the resonance parameters reported here are evaluated as7
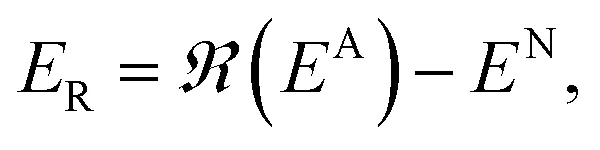
8
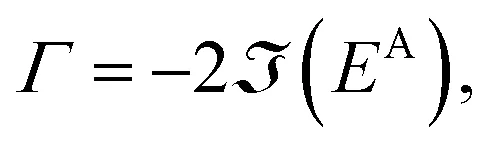
where *E*^A^ is the energy of the anionic resonance evaluated with the CAP method, and *E*^N^ is the energy of the neutral ground state obtained with a CAP-free calculation.

To ensure that the resonance parameters are converged to within meV, we use tighter convergence thresholds than the default values of Q-Chem, which we find to be insufficient for this level of precision. We set the self-consistent-field convergence threshold to 10^−12^ and both the CC and EOM convergence thresholds to 10^−10^ or 10^−9^. Although these thresholds are exceedingly tight, we prefer to be conservative nonetheless.

To achieve meV precision, we also find that *η*_opt_ sometimes needs to be more tightly converged than is commonly done. The effect is smaller than properly converging the electronic structure calculations though.

## 3 Results and discussion

### 3.1 The problem: CAP calculations yield multiple solutions

To showcase the problem, we start by revisiting the π* shape resonance of N_2_^−^. Fig. 1 shows the relevant *η*-trajectory and its associated velocity, obtained with 0th-order CAP/EOM-EA-CCSD/aug-cc-pVTZ+3s3p3d(C). In the range of *η* shown in Fig. 1, we find two CAP solutions, which we label a and b. Which is the “physical” solution? In ref. 32, resonance parameters were presented for solution a, whereas in ref. 39, resonance parameters were presented for solution b. Neither of these studies justified their choice or mentioned the existence of the other solution. This example nicely illustrates the issues we want to raise here: the appearance of multiple solutions in CAP calculations and the ambiguity in selecting one as “physical”.

**Fig. 1:**
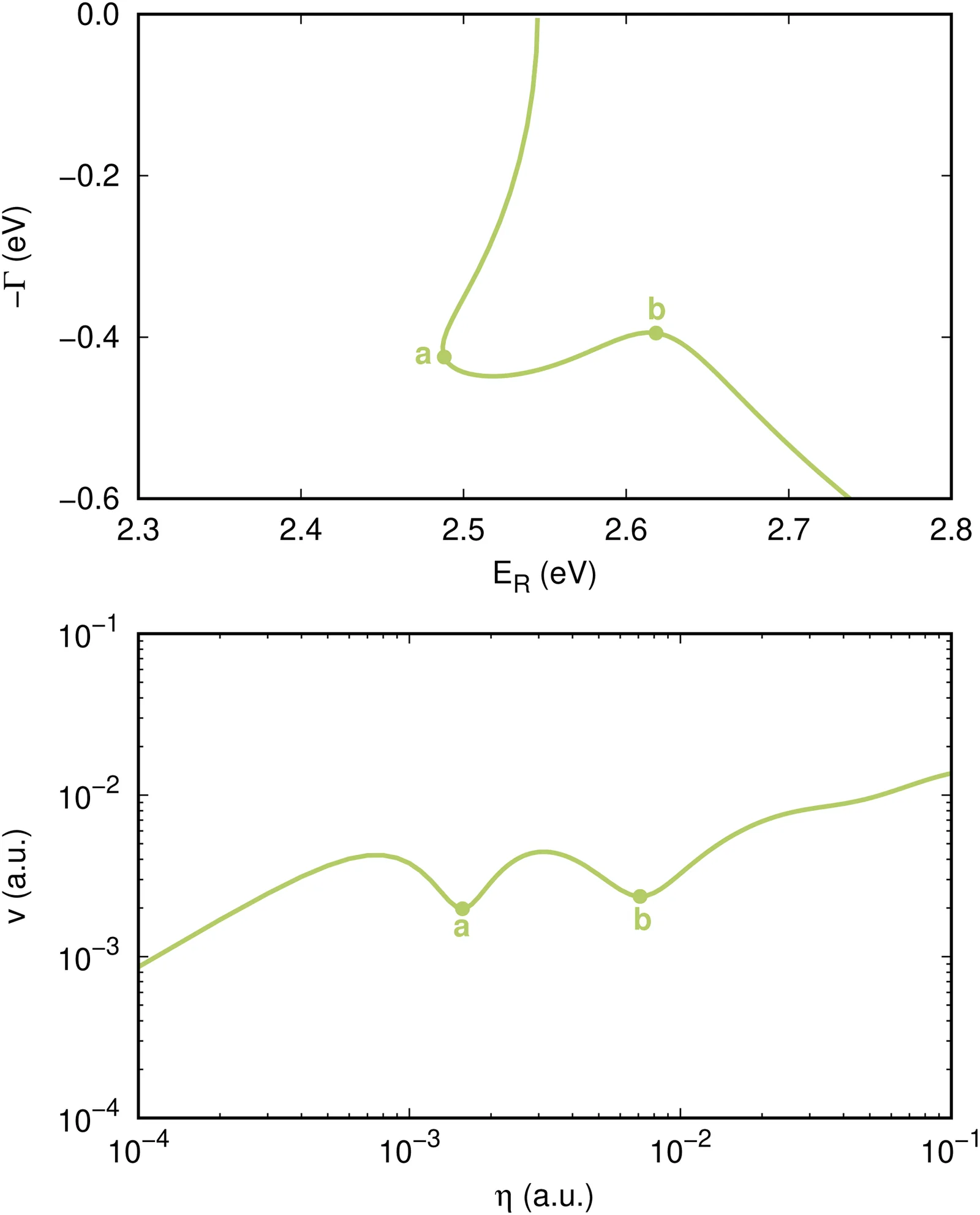
*η*-Trajectory (top) and velocity (bottom) for N_2_^−^, calculated with 0th-order CAP/EOM-EA-CCSD/aug-cc-pVTZ+3s3p3d(C). Minima a and b are marked by circles. Another minimum at higher *η* and additional *η*-trajectories are shown in Fig. 7.

Two arguments are occasionally evoked to assign a particular CAP solution to a resonance. First, it should have a small value of *η*_opt_, since *η* → 0 is the correct limit for a complete basis set. However, in a finite basis set calculation, this criterion alone is not sufficient. A solution at a smaller *η*_opt_ is less perturbed by the CAP, which is desired. But a smaller *η*_opt_ also implies a poorer description of the continuum, since the CAP may be too weak to properly absorb the wave function without causing spurious reflections arising from the finite extent of the basis set. Although reasonable, this criterion favors solutions that minimize the error from the CAP perturbation against minimizing the error from the implicit continuum, which is not necessarily preferable in a finite basis.

The second argument to select a particular CAP solution is that its velocity should be small. A low *v* indicates an improved stabilization of the resonance, and does not have the bias of the first criterion. For either criteria, however, one has to decide what is “small”, introducing another ambiguity.

Although not commonly stated, these two criteria alone are not enough to disentangle CAP solutions describing resonances and continuum-like states, as will be shown later. To do so, one needs to analyze the wave function, the third and final criterion. A useful proxy is the Dyson orbital of the CAP solution.^65^ While a continuum-like solution has a delocalized Dyson orbital, a resonance has a more localized valence-like orbital. While this is a key criterion to identify the resonant-like solutions, it still leaves room for arbitrariness, related to the degree of localization of the orbital. In summary, while the three criteria are valuable to assign a CAP solution to a resonance, they are all qualitative in nature and are often not compatible, as will also be discussed later.

Coming back to the two CAP solutions of N_2_^−^ (see Fig. 1), can these three criteria point out which one should be preferred? The first argument would favor solution a, as it is subject to a lower error due to the CAP perturbation. However, solution b has a lower error associated with the approximate description of the continuum by the CAP. There is no reason to tell which one would have lower overall error in this finite basis calculation. The second argument is also inconclusive, as the velocity of solution a is only slightly smaller. Similarly for the third argument, as both Dyson orbitals, shown in Fig. 2, have a valence-like π* character expected for the resonance, being a little more localized for solution b. Therefore, we see no basis to claim that one CAP solution would be any more physical than the other. We interpret each solution as a different way in which the CAP perturbation can stabilize the resonant wave function in a finite basis. Instead of using the above criteria to elect the “best” solution, we propose that they should guide the identification of “good” solutions, which offer an approximate description, to a greater or lesser degree, of the same physical state.

**Fig. 2:**
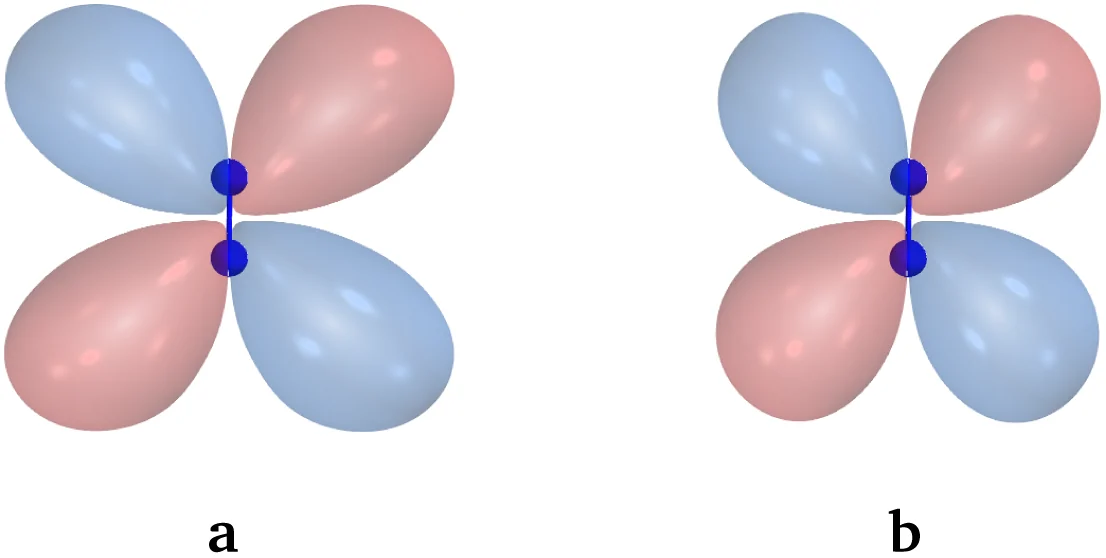
Real part of the Dyson orbital for the two minima shown in Fig. 1, labelled a and b, for an isovalue of 0.02.

The problem is not restricted to N_2_^−^. By lifting the assumption that a single CAP solution is physical, we must deal with the appearance of multiple approximate solutions. This represents an additional source of uncertainty in the determination of the resonance parameters in practical CAP calculations, which we label non-uniqueness error. The energy difference between the furthest apart admissible CAP solutions, considering real and imaginary parts separately, provides a rough estimate of this uncertainty. We consider a solution to be admissible if it (i) appears at a value of *η*_opt_ below ∼0.1–1 a.u., (ii) has a velocity below ∼10^−3^–10^−2^ a.u., (iii) is comparatively more localized, according to the localization metric defined in the next paragraph. We will come back to the non-uniqueness error for N_2_^−^ when discussing the errors due to finite basis sets, in Sec. 3.2, and the electronic structure method, in Sec. 3.3.

To further discuss the occurrence of multiple solutions, we move to the π* and σ* shape resonances of the chloroethylene anion. Our results can be seen in Fig. 3, which highlights the *η*-trajectories and associated velocities displaying solutions with localized π* and σ* characters. We identify a series of CAP solutions in addition to the single one previously reported for each symmetry.^39,44^ The real part of their corresponding Dyson orbitals is shown in Fig. 4. To quantify the character of the different solutions and help identify which ones are acceptable approximations of the resonances, we define the difference between the spatial extent of the anion and of the neutral wave functions, Δ*r* = 〈*r*^2^〉_A_^1/2^ − 〈*r*^2^〉_N_^1/2^. The expectation value of the position operator *r* is evaluated with a CAP calculation for the anion (A) and with a CAP-free calculation for the neutral (N). More localized solutions (smaller Δ*r*) are candidates to describe the resonant state, whereas more diffuse solutions (larger Δ*r*) would be indicative of continuum-like states. As we will see below, making a clear distinction is not possible in general, as the values of Δ*r* do not cluster around well-defined regimes. Still, a comparative analysis is helpful to identify the more admissible CAP solutions for the resonance. All numerical values for the CAP solutions highlighted in Fig. 3 and 4 are presented in Table S2 of the SI.

**Fig. 3:**
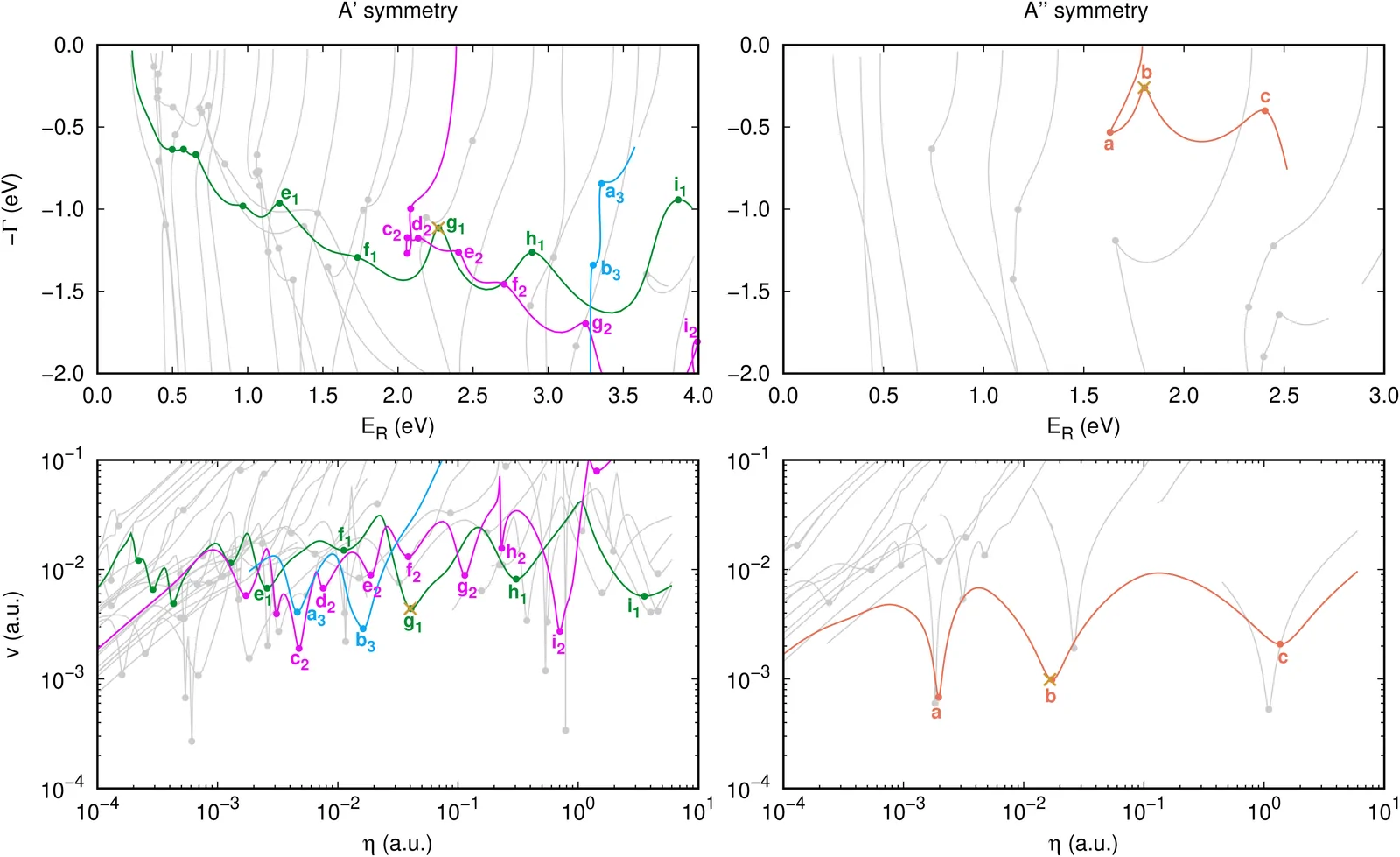
*η*-Trajectories (top) and velocities (bottom) for the *A*′ (left) and *A*″ (right) symmetries of the chloroethylene anion, calculated with 0th-order CAP/EOM-EA-CCSD/aug-cc-pVDZ+3p(A). The *η*-trajectories and associated velocities supporting solutions with localized σ* (*A*′) and π* (*A*″) characters are highlighted. Minima are marked as circles. Results from ref. 44 are marked as crosses. The real part of the Dyson orbital for selected minima are shown in Fig. 4.

**Fig. 4:**
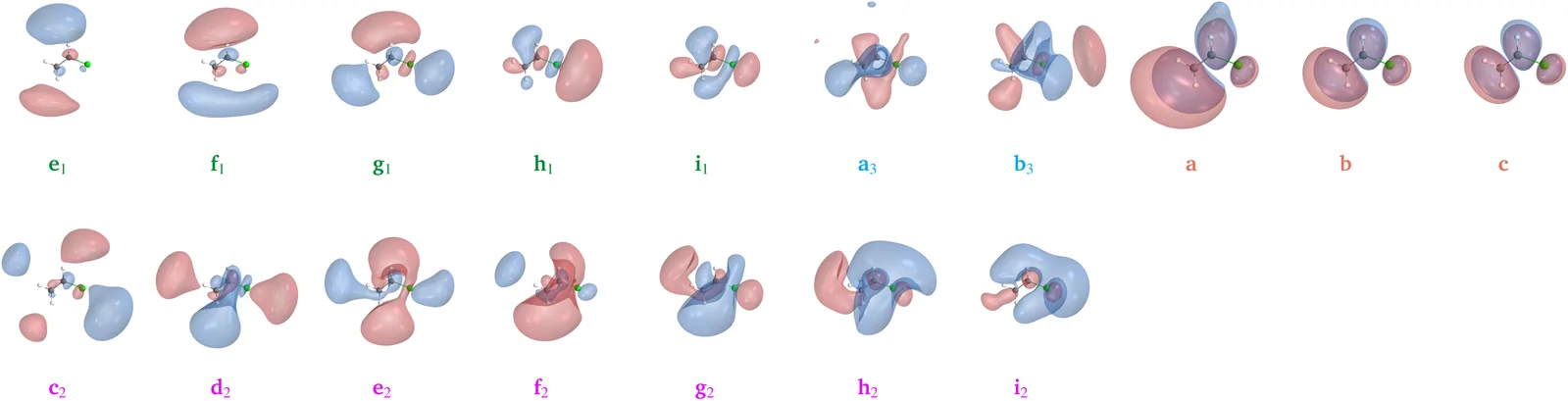
Real part of the Dyson orbital for selected minima shown in Fig. 3, identified by the same labels, for an isovalue of 0.02.

In the *A*″ symmetry, one *η*-trajectory displays three CAP solutions whose Dyson orbitals have localized π* character, as shown in Fig. 4. They are labeled a, b, and c in increasing *η*_opt_. Only solution b was reported in ref. 39 and 44 as the π* resonance. Although *η*_opt_ can be argued to be too large for solution c, its velocity is in the same order as that of solution b. In turn, solution a has both the smallest velocity and the smallest *η*_opt_. On the other hand, we notice that the Dyson orbitals become progressively more localized as *η*_opt_ increases, thus acquiring a more resonant-like character, with Δ*r* of 1.0 Å (a), 0.8 Å (b), and 0.6 Å (c). Faced with these ambiguities, both solutions a and b can be taken as acceptable approximations of the π* resonance, whereas solution c is probably too perturbed by the CAP. We thus assign a non-uniqueness error of 0.17 eV for the position and 0.27 eV for the width.

The situation for the σ* resonance is even more serious. As shown in Fig. 3, there is a plethora of minima scattered throughout the complex plane. The single minimum identified as the σ* resonance in ref. 39 and 44 is labeled as g_1_ here. Clearly, its values of *η*_opt_ and velocity bear no special significance. The usual arguments that the physical solution would have the smallest values of *η*_opt_ or of the velocity would have pointed to other CAP solutions. Therefore, this particular solution must have been elected from an analysis of its wave function. Indeed, we find that its Dyson orbital has a relatively localized σ* character (see Fig. 4). However, and crucially, the σ* character is not exclusive to this CAP solution, while its degree of localization, of Δ*r* = 1.4 Å, is not special either. Along the same *η*-trajectory, solutions h_1_ and i_1_ have an even more dominant and localized σ* character, with Δ*r* of 1.0 Å and 0.6 Å, while solutions e_1_ and f_1_ have a p-type Rydberg character, with Δ*r* of 2.0 Å and 1.8 Å, not much larger than that of g_1_. In fact, solution g_1_ falls in the middle with a mixed σ* and p-type Rydberg character and an intermediate degree of localization. One could argue that its Dyson orbital is not sufficiently localized in comparison to those of solutions h_1_ or i_1_, which have comparable velocities but perhaps too large values of *η*_opt_. The ambiguity does not end there, as two other *η*-trajectories display solutions with important σ* admixture and comparable degrees of localization. Solutions c_2_ to i_2_ also have a Rydberg character shifting from d- to s-type as *η*_opt_ increases. Although solutions c_2_ and d_2_ are more diffuse, with Δ*r* of 2.1 Å and 1.8 Å, they already display some σ* character and appear at small *η*_opt_. Solutions e_2_ and f_2_ acquire a more mixed character and an intermediate degree of localization, with Δ*r* of 1.4 Å and 1.5 Å. They are thus comparable to the case of solution g_1_ (1.4 Å), all occurring at a similar range of *η*_opt_, though with higher velocities. The next solution along this *η*-trajectory, g_2_, is considerably more localized (0.8 Å) while its *η*_opt_ and velocity are somewhat larger than those of g_1_. Solutions h_2_ and i_2_ are also very localized (0.7 Å and 1.0 Å), but are more strongly perturbed by the CAP. The third *η*-trajectory with an important σ* character has two solutions, a_3_ and b_3_. In comparison to solution g_1_, both have smaller *η*_opt_, and comparable velocities and degree of localization, of 1.5 Å (a_3_) and 1.4 Å (b_3_). Trying to fulfill the three criteria discussed in Sec. 3.1 (small values for *η*_opt_, velocity, and Δ*r*), we take the CAP solutions g_1_, h_1_, g_2_, a_3_, and b_3_ as the most reasonable approximations of the σ* resonance.

In summary, besides the solution g_1_,^39,44^ the other CAP solutions identified here have a comparable degree of σ* character and velocities on the same order of magnitude, although values of *η*_opt_ that can be considerably smaller or larger. Once again, we find no basis to support that the specific solution previously identified^39,44^ would be more physical than some of the others. Estimating a non-uniqueness error is equally prone to ambiguities, depending on which CAP solutions are deemed acceptable. Taking the five CAP solutions pointed out above, we assign a non-uniqueness error for the σ* resonance of chloroethylene of 1.1 eV for its position and of 0.9 eV for its width. This is significantly more than for the π* resonance, and comparable to what would be estimated from a conventional electronic structure calculation that neglects the continuum. Despite the large non-uniqueness error for the σ* resonance, it is interesting to note that, while the previously identified solution g_1_ (ref. 39 and 44) underestimates the experimental position (2.84 eV)^66,67^ by 0.5 eV, the other CAP solutions identified here either approach (h_1_) or overestimate (h_1_, a_3_, b_3_) it by a comparable amount of 0.4 eV to 0.5 eV.

Based on our example for the chloroethylene anion, we suspect that short-lived resonances, often of σ* character, may be subject to larger non-uniqueness errors. Resonances having shorter lifetimes are more strongly coupled with continuum states, which are only implicitly described by the CAP. Disentangling continuum and short-lived resonant states may thus be more challenging with current CAP calculations, perhaps causing larger non-uniqueness errors.

Besides the current and the previous^43,44^ CAP/EOM-EA-CCSD calculations, other studies have relied on different levels of theory in combination with the CAP method to describe the anionic resonances of chloroethylene: CAP/ADC(2),^55^ CAP/SAC-CI,^34^ and CAP/XMS-CASPT2.^34^ Since all have reported a single solution for each state and no *η*-trajectories, it is not possible to say whether there is a correspondence between the different solutions, as will be discussed for N_2_^−^ in Sec. 3.2. This exemplifies how limited information on CAP calculations and the assignment of their solutions prevents a proper comparison among different electronic structure methods. Yet, we notice that the reported resonance parameters (see Table S3 of the SI) vary within 0.2 eV for the π* resonance, and within 0.4 eV for the σ* resonance, which are comparable (π*) or less (σ*) than the CAP/EOM-EA-CCSD non-uniqueness error for the position of π* (0.17 eV) and σ* (1.1 eV) resonances. In turn, these errors are significantly larger than the experimental uncertainty of 0.03 eV.^67^

The *η*-trajectories seen in Fig. 3 also reveal an interesting pattern. Most of the solutions are located along an approximately linear band that starts at *E*_R_ = 0 and *Γ* = 0. The pattern can be hinted in the *A*″ symmetry, but is more evident in the *A*′ symmetry. It is reminiscent of complex scaling, where the continuum-like solutions appear rotated with respect to the real axis by a fixed angle.^10,11,68^

In the next four subsections, we investigate how the CAP solutions are affected by different aspects of the calculation.

### 3.2 The role of the electronic structure method

Starting with the role of electronic correlation, Fig. 5 gathers results for N_2_^−^ obtained with the CAP method in combination with EOM-EA-CCSD, ΔUCCSD, and ΔUHF (present work), MRCI with singles (MRCIS),^51^ qsGW,^38^ and full configuration interaction (FCI),^36^ the last obtained at the low-*η*_opt_ solution of CAP/EOM-EA-CCSD. They were all obtained for the same geometry, CAP onsets, and the aug-cc-pVTZ+3s3p3d(C) basis set. This offers the rare possibility of assigning the observed differences exclusively to the description of electronic correlation. The numerical values can be found in Table S4 of the SI.

**Fig. 5:**
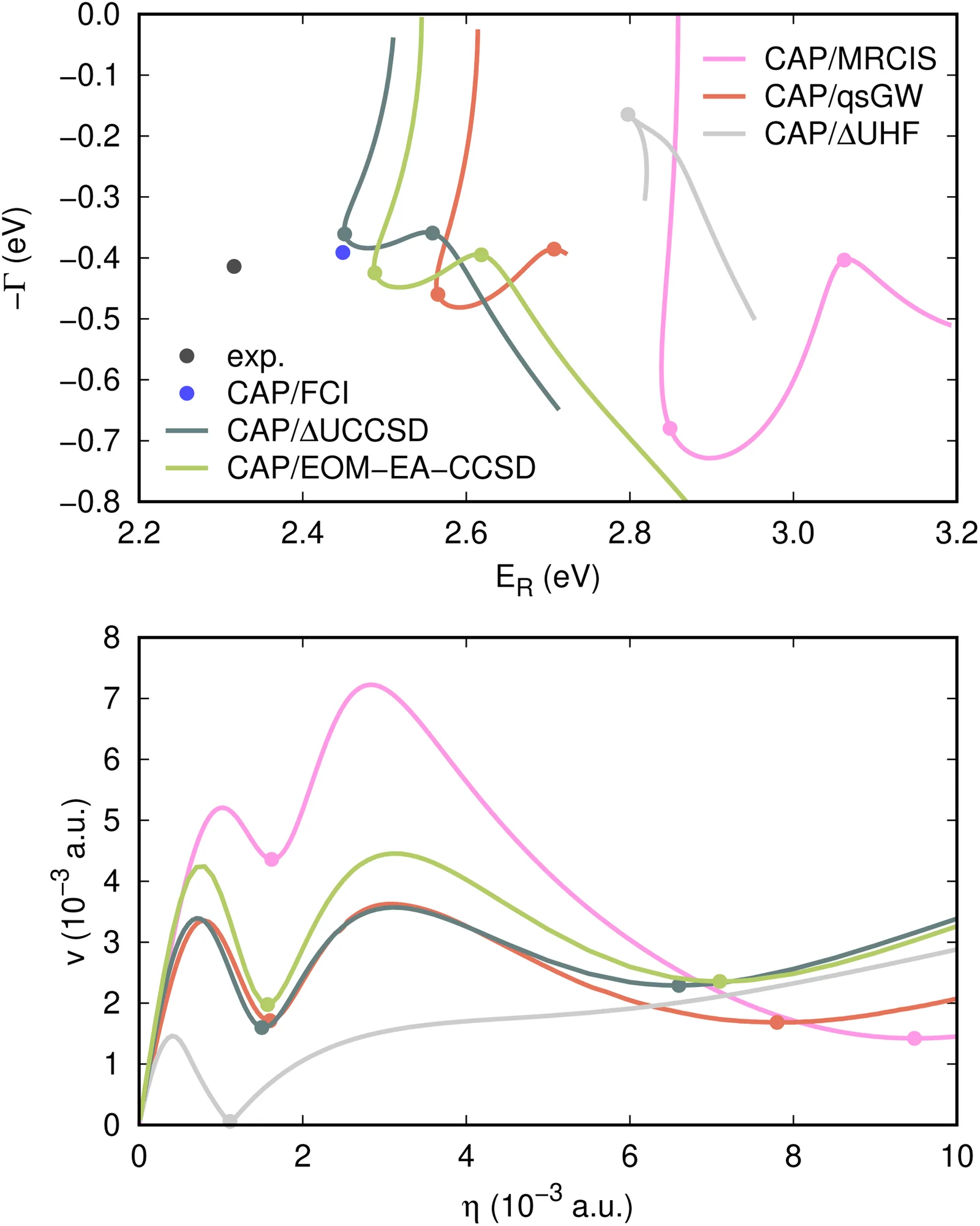
*η*-Trajectories (top) and velocities (bottom) for N_2_^−^, calculated for the same geometry, CAP onsets, and basis set [aug-cc-pVTZ+3s3p3d(C)], but different electronic structure methods: CAP/EOM-EA-CCSD (same as in Fig. 1), CAP/ΔUCCSD, and CAP/ΔUHF (present work), CAP/qsGW,^38^ CAP/MRCIS,^51^ and the CAP/FCI single point obtained at the low-*η*_opt_ solution of CAP/EOM-EA-CCSD.^36^ Minima are marked as circles. The experimental estimate is from ref. 69.

For all correlated methods, the *η*-trajectories and associated velocities are remarkably similar. The same two minima are observed in all cases at comparable values of *η*_opt_. Although the CAP/MRCIS results^51^ show larger deviations, reflecting its more approximate description of correlation, the trajectory and velocity profiles are still similar to those of the other correlated methods. Even the CAP/ΔUHF mean-field description is able to support the low-*η*_opt_ minimum, whereas the high-*η*_opt_ minimum falls short of being stabilized. Our results demonstrate that the emergence of multiple solutions in CAP calculations is not related to the description of electronic correlation.

Comparing the two methods that treat correlation more extensively, we find that CAP/ΔUCCSD yields a low-*η*_opt_ solution that is somewhat closer to the CAP/FCI reference theoretical value^36^ than CAP/EOM-EA-CCSD, with an absolute difference of only 0.03 eV, compared to 0.05 eV. In turn, the absolute energy gap between the two CAP solutions is significantly larger, at 0.11 eV (ΔUCCSD) and 0.13 eV (EOM-EA-CCSD). Also for the CAP/qsGW method,^38^ the absolute difference with respect to CAP/FCI (0.13 eV) remains below the gap between its two CAP solutions (0.16 eV). Only for the more approximate CAP/MRCIS method the absolute difference to CAP/FCI (0.49 eV) surpasses the difference between its two solutions (0.35 eV), although both are comparable. For all methods, the non-uniqueness error is larger or at least comparable to the error from the approximate electronic structure. In absolute terms, it decreases as the method becomes more accurate. In contrast, the relative importance of the non-uniqueness grows for the more accurate methods.

The most reliable experimental estimate^69^ for the vertical resonance energy of N_2_^−^ is also indicated in Fig. 5. It was obtained by fitting measured vibrational excitation cross sections to a theoretical model. Although no uncertainty was reported,^69^ the spacing of the data suggests an experimental uncertainty of around 0.03 eV, on par with similar experiments. This is less than the non-uniqueness error of CAP calculations, even for the most accurate electronic structure methods, ΔUCCSD (0.11 eV) and EOM-EA-CCSD (0.13 eV).

Interestingly, the *η*-trajectory obtained with CAP/ΔUHF starts at *η* = 0 with a non-zero imaginary part (see Fig. 5), and not at the real axis, as would normally be expected. This means that the UHF *η*-trajectory that best describes the N_2_^−^ resonance is not connected with a usual (real) UHF solution of the CAP-free problem, but rather with a symmetry-broken complex-valued Hartree–Fock solution. We believe this has never been reported before.

At the CAP/ΔUCCSD level, a complex-valued energy is still obtained at *η* = 0, though much closer to the real axis. The inclusion of electronic correlation helps to restore the symmetry that has been broken at the mean-field level, similarly to what is known for bound-state calculations.

### 3.3 The role of the basis sets

It has been stated that too small basis sets in CAP calculations would give rise to spurious minima in eqn (5), accounting for the multiple CAP solutions.^70^ This claim referred to previous works,^32,56,71^ of which only one^32^ explored different bases, although multiple solutions or their dependence on the choice of basis set were not mentioned. Therefore, the role of basis set on the appearance of multiple minima remains unexplored to the best of our knowledge.

To address this point, here we report an extensive basis set investigation for N_2_^−^, focusing on the structure of multiple minima. Fig. 6 gathers the *η*-trajectories and velocities obtained with 0th-order CAP/EOM-EA-CCSD, for ten different basis sets. All CAP solutions maintain the localized π* character that is expected for the resonance, as illustrated for the aug-cc-pV5Z+3s3p3d(A) basis set in Fig. S2 of the SI. The numerical values of all CAP solutions are presented in Table S5 of the SI. The results for the aug-cc-pVTZ+3s3p3d(C) basis set are the same as shown in Fig. 1. The aug-cc-pVXZ+3s3p3d(A) basis sets, where X = D, T, Q, have been recently employed,^54^ although a single CAP solution has been reported for each.

**Fig. 6:**
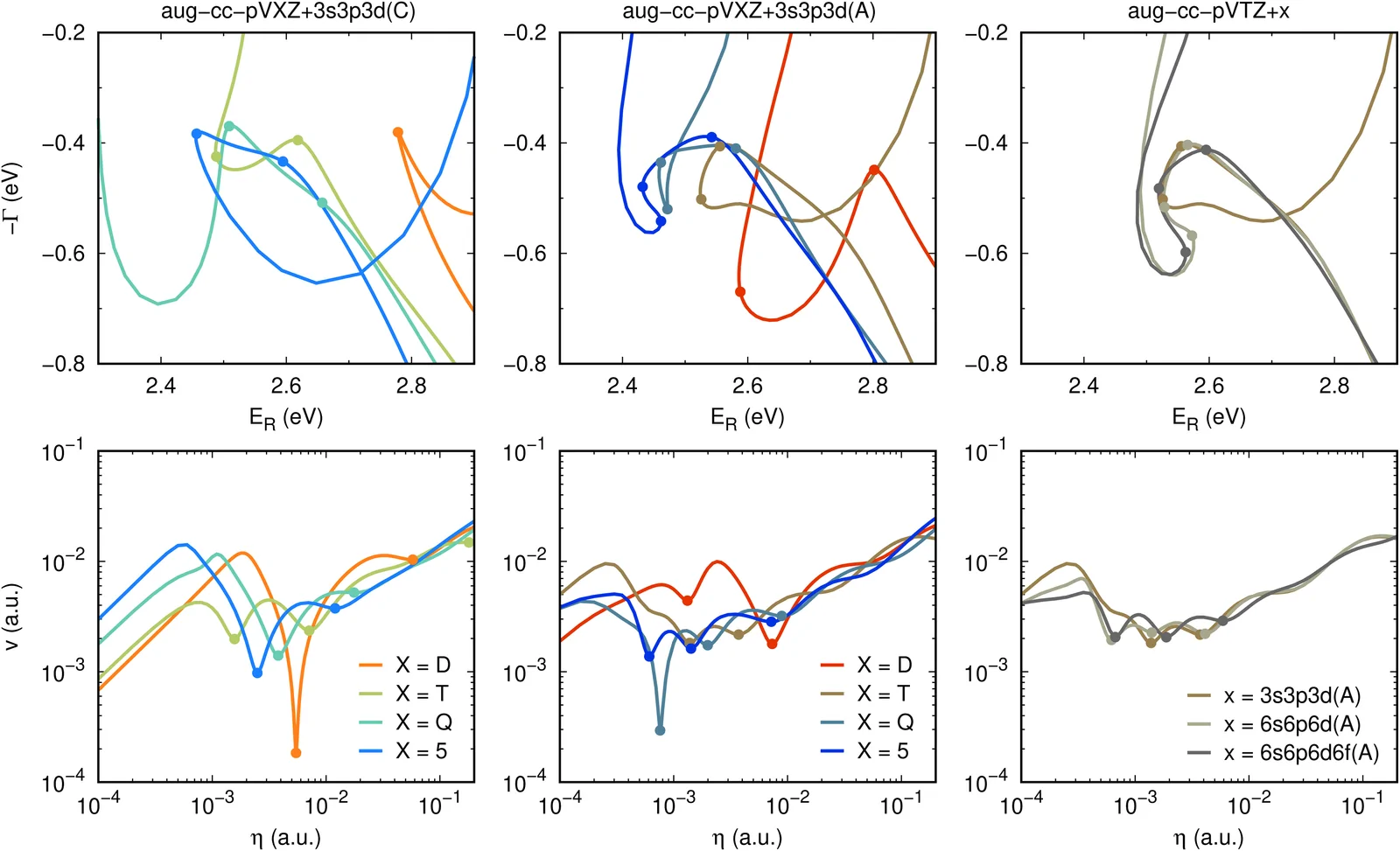
*η*-Trajectories (top) and velocities (bottom) for N_2_^−^, calculated with 0th-order CAP/EOM-EA-CCSD and various basis sets. Minima are marked as circles. For a variable valence set (*X*) increasing from double-zeta to quintuple-zeta, the extra set of 3s3p3d functions is placed either at the geometric center of the molecule (left column) or at each atom (middle column), or, on top of the aug-cc-pVTZ basis set, the extra set of functions (*x*) increases from 3s3p3d(A) to 6s6p6d6f(A) (right column). The results for the aug-cc-pVTZ+3s3p3d(C) basis set (left column, green line) are the same as in Fig. 1.

There are many important observations to make from Fig. 6. We find that the number of multiple solutions is essentially independent of the basis set, oscillating between two and three in the energy range shown in Fig. 6. Our results thus contradict the statement that the multiple minima would be a consequence of too small basis.^70^ In fact, they persist for basis sets that are very large in the valence region [aug-cc-pV5Z+3s3p3d(A)] and in the diffuse region [aug-cc-pVTZ+6s6p6d6f(A)].

We also find no correlation between the energy range spanned by the multiple CAP solutions and the size of the basis set. Based on the difference between the furthest pair of CAP solutions, the non-uniqueness error oscillates between 0.1 eV and 0.2 eV. The two double-zeta bases are the exception, with an error reaching 0.3 eV (A) or having a single low-velocity solution (C). Across different bases, the non-uniqueness error is larger than the apparent experimental uncertainty of around 0.03 eV.^69^ It seems very unlikely that further augmenting the basis as is customarily done would make the solutions to cluster around a smaller area. It is also interesting to notice that the size of the basis set does not correlate with the lowest value of the velocity.

How does the non-uniqueness error compare with the basis set incompleteness error? Increasing the valence basis set completely changes the structure of CAP solutions, making it impossible to track the convergence for a particular solution. However, we observe that the average energy of the multiple solutions change progressively less as the basis increases, which is more apparent for the aug-cc-pVXZ+3s3p3d(A) sequence (top middle panel of Fig. 6). Taking the average energy of the CAP solutions, and the quintuple-zeta basis as the reference, we estimate basis set errors decreasing from 0.24 eV (double-zeta) to 0.07 eV (triple-zeta) and 0.03 eV (quadruple-zeta). Except for the smallest basis, these values are comparable to the error associated with the electronic structure method, of 0.05 eV for CAP/EOM-EA-CCSD (see Sec. 3.2), both being smaller than the non-uniqueness error (0.1 eV to 0.2 eV). Therefore, the existence of multiple solutions represents the main source of uncertainty in CAP calculations for the resonance energy of N_2_^−^, provided that an accurate level of theory and a reasonably sized basis are chosen.

Augmenting the extra diffuse set from 3s3p3d(A) to 6s6p6d(A) produces small oscillations in the velocity profile and the resonance parameters, of around 0.01 eV. An undulation at low *η* is stabilized into a new solution, though. Further augmenting with diffuse functions of higher angular momentum [6f(A)] causes somewhat larger shifts on the energies, of around 0.03 eV, and on the velocity, although the structure of minima is largely preserved. At this stage, there is some degree of saturation on the resonance parameters, where multiple solutions still exist. Their persistence shows that the standard procedure to generate even-tempered bases is inadequate to describe resonance states as a single CAP solution.

Despite the radial and angular saturation we just discussed, placing the extra diffuse set on the center-of-mass (C) or on each atom (A) produces qualitatively different structure of minima. Moreover, the latter option yields lower resonance positions and larger widths, by 0.06 eV and 0.08 eV, on average. This reveals an additional problem of even-tempered basis in CAP calculations: the non-negligible dependence on their center. Furthermore, there are generally more minima with the larger, atom-centered bases, although the non-uniqueness error does not change much.

Comparing our 0th-order and 1st-order results (see Fig. S3 and Table S6 of the SI) reveals interesting similarities and differences. The main conclusions discussed above also hold for the 1st-order results: the number of minima does not depend on the basis set size, there is no evidence of basis set convergence, the non-uniqueness errors are larger than the other sources of uncertainty, and even-tempered basis sets are deficient.

In contrast, the structure of 1st-order CAP solutions span a substantially larger energy range. Averaging the non-uniqueness error over the ten bases considered here, and restricted to *η*_opt_ < 0.2 a.u., yields 0.16 eV (0th-order) and 0.39 eV (1st-order). Furthermore, the number of solutions often increases when going from 0th- to 1st-order, from 2.5 to 3.3 on average. Both observations are independent on the size of the basis. Another interesting difference is that the minima are generally deeper and sharper in the 1st-order results, independently of their *η*_opt_ and the basis, indicating an improved stabilization of the various CAP solutions, even though they are more numerous and spread in energy.

### 3.4 The roles of the optimization criterion and CAP shapes

While the stationarity condition for the velocity [eqn (5)] is the most popular protocol for optimizing *η*, other ideas have been proposed. One is to take separate values of *η* that minimize the real and the imaginary components of the velocity,^56^ although this is rarely done. Clearly, this criterion does not change the scenario of multiple solutions.

Another possibility is to perform an analytical continuation of the *η*-trajectory toward *η* → 0.^57,58^ This is done by discarding the low-*η* segment of the *η*-trajectory, for which the resonance would not yet be sufficiently stabilized by the CAP. This segment corresponds to the region below a given minimum in the standard optimization protocol [eqn (5)]. The problem, of course, is that one has to choose a reference minimum. Different choices lead to different analytical continuations and thus resonance parameters. The existence of multiple solutions cannot be fixed with the analytical continuation technique.

More recently, an alternative criterion has been proposed to optimize the CAP, where one minimizes the error function^59^9
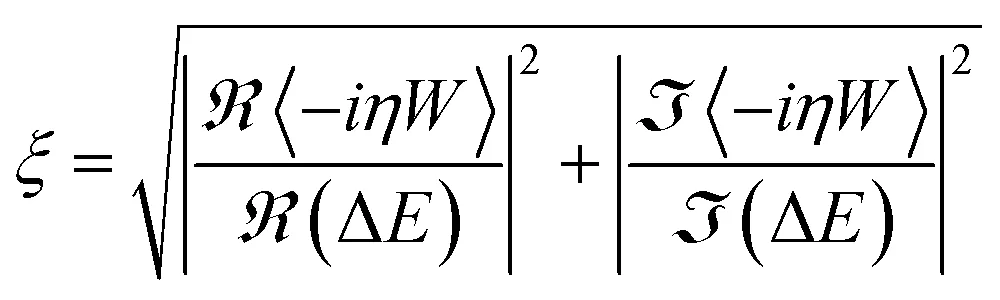
Δ*E* being the (complex-valued) energy of the anionic resonance minus the (real-valued) energy of the neutral.

Here, we explore the structure of minima of the function *ξ*. This criterion has been employed to optimize the CAP onset for a fixed CAP strength *η*,^59^ while here we use it to optimize *η* for a fixed onset. This allow us to directly compare the minima of *ξ* and of the velocity. While some resemblance between the profiles of the two functions has been briefly mentioned before,^59^ the present results reveal additional similarities and key differences.

The comparison for N_2_^−^ can be seen in Fig. 7 for the aug-cc-pVTZ+3s3p3d(C) basis set, and in the SI for various other basis sets (Fig. S4) and also for the two symmetries of the chloroethylene anion (Fig. S5). In most cases, minima of the velocity have a direct counterpart in the *ξ* function. However, the latter supports additional minima, making it even more prone to the problem we have been discussing. As an example, the average number of CAP solutions for N_2_^−^ (over the ten bases considered here and with *η*_opt_ < 0.2 a.u.) increases from 2.5 according to the 0th-order velocity criterion to 3.8 according to the *ξ* criterion. For the minima that appear in both functions, the resonance parameters vary little, within 0.03 eV, but typically less.

**Fig. 7:**
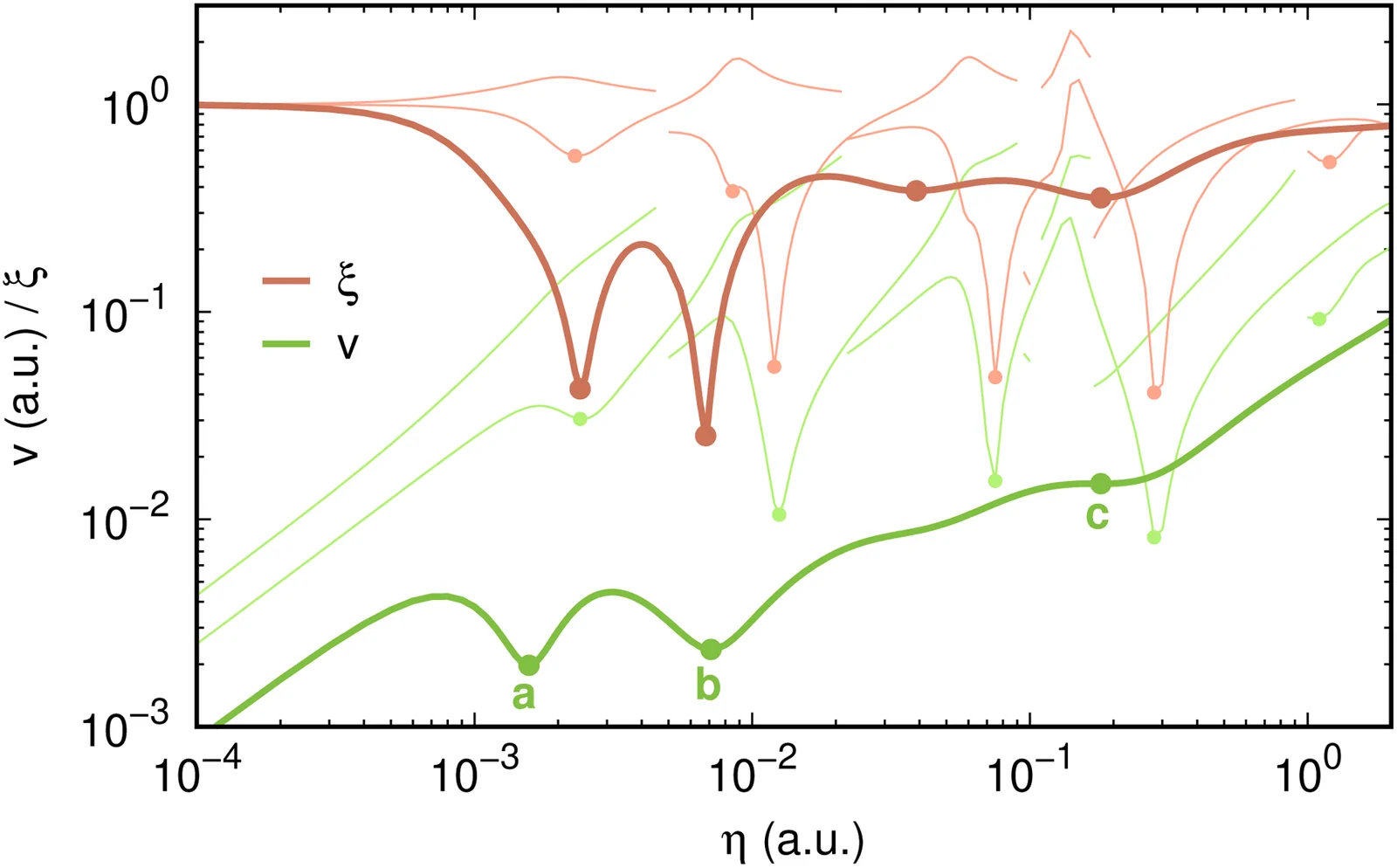
The velocity [eqn (5)] and the *ξ* function [eqn (9)] for N_2_^−^, obtained with 0th-order CAP/EOM-EA-CCSD/aug-cc-pVTZ+3s3p3d(C). Thick lines correspond to the *η*-trajectory supporting solutions with localized π* character, and thin lines correspond to other *η*-trajectories. Minima are marked as circles. The velocity supports three minima, labeled a, b, and c, the former two being the same as in Fig. 1.

The additional minima in *ξ* can be partially explained by its flatter behavior, which in turn would be destabilized thanks to the *η* linear term in the expression of the velocity [eqn (3)]. This accounts for the stabilization of the additional minimum seen in Fig. 7 for N_2_^−^, for instance. This is the exception, though. In most cases, the additional minima in *ξ* do not correspond to regions of near stability of the velocity, and are typically shallower than those that appear in both functions. We also notice that the minima in *ξ* are overall narrower and deeper than the analogous minima in the velocity. As already discussed for the velocity, we find no relation between the lowest value of *ξ* and the size of the basis set.

We further explore how the multiple minima of N_2_^−^ are affected by the shape of the CAP. Besides the cuboid CAP, we used a spherical and a smooth Voronoi CAP,^61,62^ the latter two with the previously used onset of 4 a_0_.^34^ While the velocities and *η*-trajectories are rather similar (see Fig. S6 of the SI), in line with previous findings,^61,72^ our key observation is that the three shapes yield the same structure of minima. Their resonance parameters oscillate within 0.03 eV, mostly due to the imaginary part (see Table S7 of the SI). Despite the different CAP shapes, here we have employed comparable CAP onsets.

Very recently, Sanov relied on the *ξ* function to propose a new optimization strategy where both the CAP onsets and the CAP strength *η* are simultaneously optimized.^63^ The first applications, to N_2_^−^ and CO^−^, yielded considerably different onsets than those usually adopted (based on the spatial extent of the neutral wave function) and significant shifts to resonance parameters, between 0.1 eV and 0.2 eV.^63^ The structure of minima that we have been discussing here are one-dimensional cuts of a two-dimensional space explored by Sanov, featuring *η* and a single CAP onset as degrees of freedom. Interestingly, a single minima was reported in this expanded space, for both N_2_^−^ and CO^−^,^63^ suggesting that the multiple solutions could be an artifact of using fixed CAP onsets. However, the two-dimensional results feature multiple and intricate branches which could in principle support additional minima. A key and open question, which goes beyond the scope of the present work, is how the optimization of the CAP onsets affect the appearance of multiple solutions.

To summarize, the CAP onset^63^ and the basis set (Sec. 3.2) emerge as the key ingredients that determine the structure of multiple minima in CAP calculations, whereas the electronic structure method (Sec. 3.3), the particular optimization criterion, and the shape of the CAP (Sec. 3.4) all play smaller roles.

### 3.5 The role of adding a real part to the CAP

The continuum-remover potential method^50^ has been proposed as a technique to remove the continuum-like or “unphysical” solutions of CAP calculations. It consists in adding a real component *λŴ* (the so-called continuum-remover) to the purely imaginary absorbing potential of the CAP, making the perturbation truly complex:

10 *Ĥ*(*η*,*λ*) = *Ĥ*_0_ + (*λ* − *iη*)*Ŵ*

In its first application, gradually increasing *λ* was shown to make some of the CAP solutions in He and H_2_ to disappear.^50^ They have been interpreted as unphysical, whereas one of the remaining minimum, less affected by *λ*, has been assigned as physical.^50^ In a second study employing this technique, CAP/MRCIS calculations yielded two minima for N_2_^−^.^51^ While the low-*η*_opt_ minimum disappeared when *λ* was increased, the high-*η*_opt_ minimum remained, which was interpreted as the physical solution.^51^

Our results support a different picture. Rather than removing unphysical solutions while preserving the physical one,^50,51^ we show that introducing a real part to the CAP removes all solutions, regardless of their character, as long as its strength (*λ*) becomes comparable to that of the imaginary absorbing potential (*η*).

The argument is illustrated with our results for the chloroethylene anion, shown in Fig. 8, which highlights the *η*-trajectories containing the most minima with localized π* and σ* characters. For both symmetries, as the real perturbation *λ* is turned on, the minimum having the smallest *η*_opt_ is destabilized and then vanishes, whereas the higher-lying minima are virtually unperturbed. Further increasing *λ* causes the minimum having the second smallest *η*_opt_ to vanish, and so on. For sufficiently large *λ*, even the minimum having the largest *η*_opt_ disappears. We find that every CAP solution undergoes a transition between a stability region (*λ* ≪ *η*_opt_), passing through a destabilizing regime (*λ* ≈ *η*_opt_), into vanishing (*λ* ≫ *η*_opt_). As *λ* increases, the velocity profile becomes progressively flatter, covering a larger range of *η*, and destabilizing all minima along the way.

**Fig. 8:**
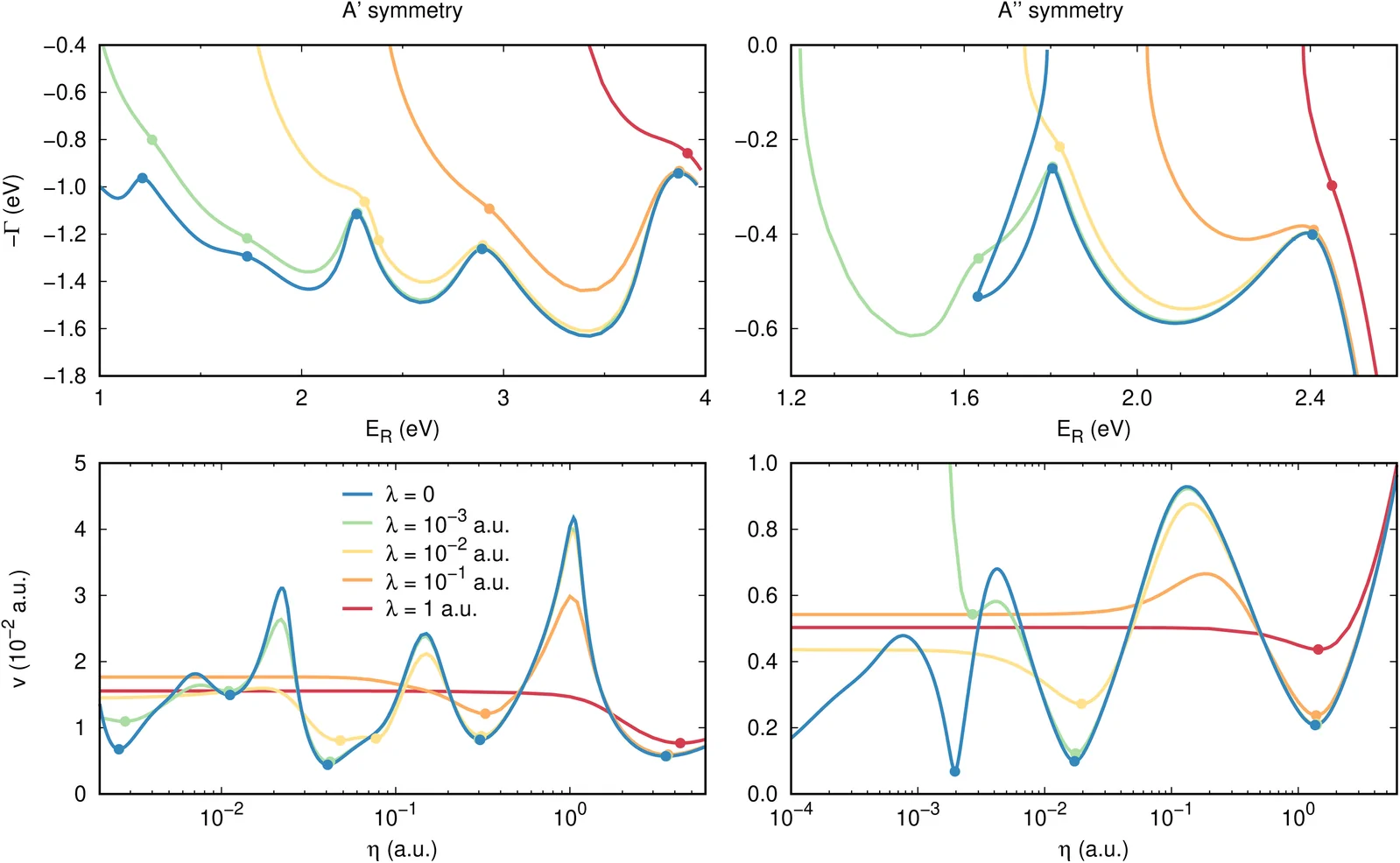
*η*-Trajectory (top) and velocity (bottom) for the *A*′ (left) and *A*″ (right) symmetries of the chloroethylene anion, supporting solutions with localized σ* and π* characters, respectively, calculated with 0th-order CAP/EOM-EA-CCSD/aug-cc-pVDZ+3p(A), for five different values of *λ*, defined in eqn (10). Minima are marked as circles. Results for *λ* = 0 are the same as those shown in Fig. 3 in green (*A*′) and orange (*A*″).

We do not observe any particularity for one of the minima. On the contrary, they all undergo the same transition. This behavior is observed for the minima appearing in other *η*-trajectories too, independently of their dominant character (resonant-like or continuum-like), as can be seen in Fig. S7 and S8 of the SI. Therefore, the continuum-remover technique^50^ cannot distinguish between resonant- and continuum-like solutions, or between what has been referred to as physical and unphysical.

The same can be concluded from the analogous results for N_2_^−^, shown in Fig. 9: increasing *λ* causes both minima to vanish, first the low-*η*_opt_ minimum and then the high-*η*_opt_ minimum. This does not mean that the former would be more physical than the latter, in contrast to the interpretation of ref. 51.

**Fig. 9:**
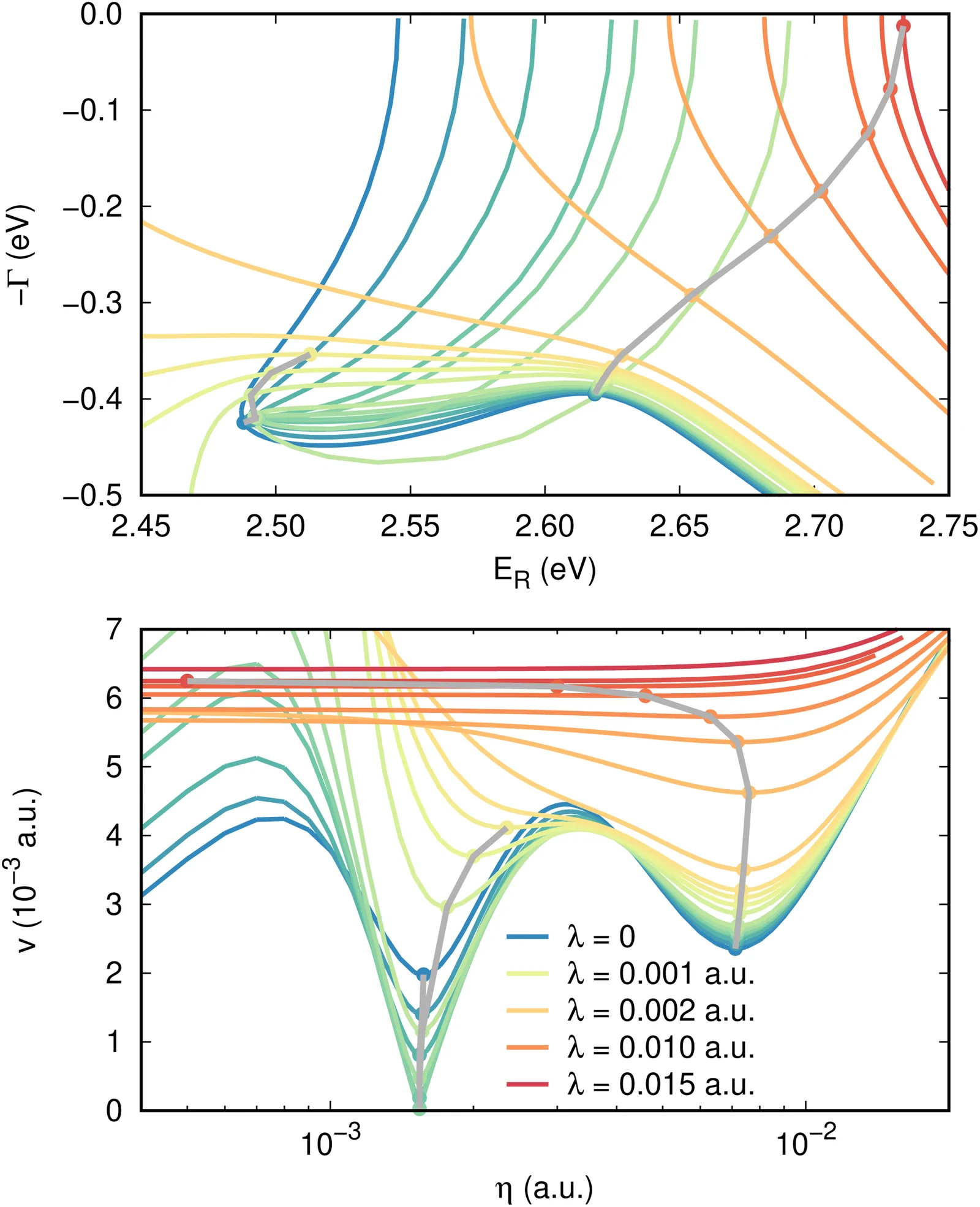
*η*-Trajectories (top) and velocities (bottom) for N_2_^−^, calculated with CAP/EOM-EA-CCSD/aug-cc-pVTZ+3s3p3d(C), for various values of *λ*, defined in eqn (10). The gray lines track the two minima. Results for *λ* = 0 are the same as those shown in Fig. 1.

Interestingly, when the real potential is turned on, the low-*η*_opt_ minimum of N_2_^−^ is initially stabilized before being destabilized. As for the high-*η*_opt_ minimum, it is significantly displaced towards smaller *η* as *λ* increases. At *λ* = 0.01362 a.u., the minimum is very close to the real axis, and the velocity derivative at *η* = 0 is negative. At *λ* = 0.01363 a.u., the minimum is no longer visible, and the velocity derivative at *η* = 0 is positive. Therefore, for an intermediate value of *λ*, the velocity must be stationary at *η* = 0, meaning that the CAP solution appears exactly at the real axis, vanishing for larger *λ*. Although we did not follow the minima of chloroethylene as closely, it seems likely that some of them also move towards and disappear at the real axis.

### 3.6 CAP calculations do not yield smooth potential energy surfaces

So far, the discussion has focused on single-point energy calculations. How does the structure of CAP solutions depend on the molecular geometry? The case of N_2_^−^ is specially interesting, as smooth potential energy curves have been reported in previous CAP calculations.^39,51,64^ In line with the equilibrium geometry results (see Sec. 3.1), solution a was elected in ref. 64, whereas solution b was elected in ref. 39, despite both using the same methodology. The latter was also selected in ref. 51, which was justified by the instability of solution a under a real perturbation (see, however, our findings in Sec. 3.5). The fact that the three studies have reported smooth curves suggests that both solutions a and b would remain stable upon bond stretching, and therefore that the structure of CAP solutions would depend weakly on the geometry. Our results demonstrate the opposite.

To revisit the potential energy curves of N_2_^−^, we employ the same methodological details as in ref. 64. Our 0th-order and 1st-order results are presented in Fig. 10 and 11, respectively, together with the data from ref. 64.

**Fig. 10:**
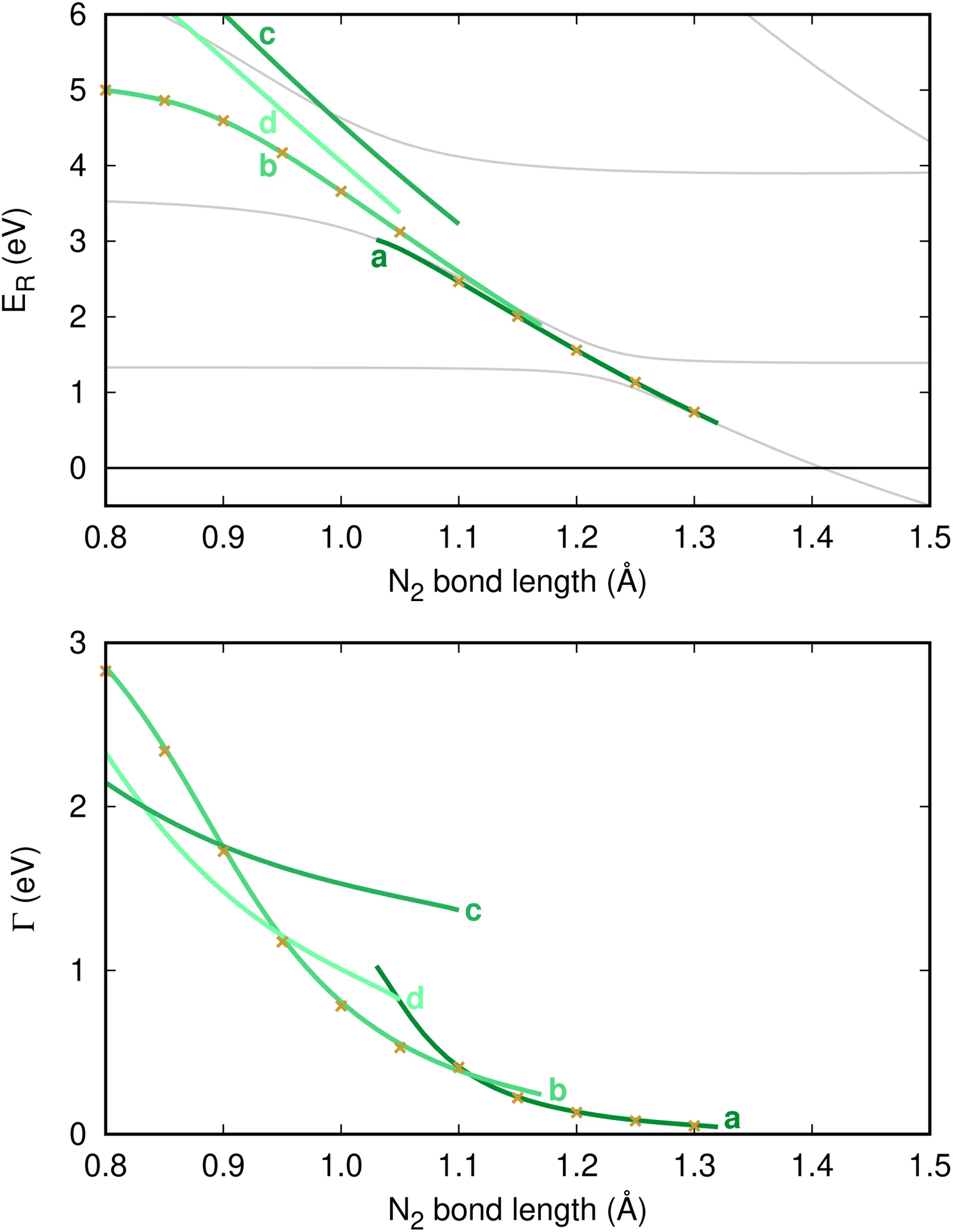
The resonance position (*E*_R_) and width (*Γ*) of N_2_^−^, calculated with 0th-order CAP/EOM-EA-CCSD/aug-cc-pVTZ+3s3p3d(C), display multiple solutions that appear and disappear as the bond length varies. The gray curves denote the CAP-free solutions. Results from ref. 64 are marked as crosses. At the equilibrium geometry of N_2_, around 1.10 Å, solutions a, b, and c correspond to those shown in Fig. 7.

**Fig. 11:**
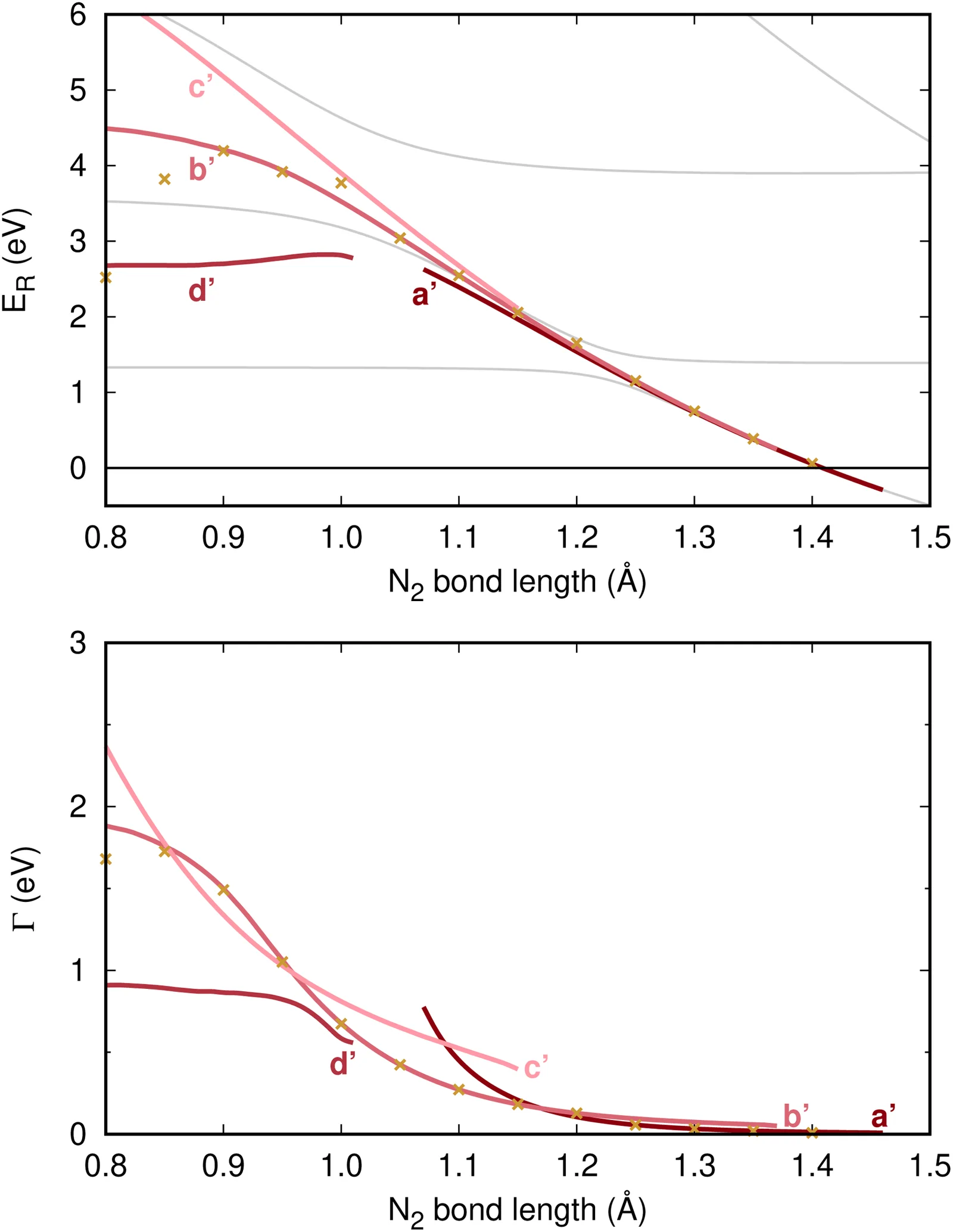
The resonance position (*E*_R_) and width (*Γ*) of N_2_^−^, calculated with 1st-order CAP/EOM-EA-CCSD/aug-cc-pVTZ+3s3p3d(C), display multiple solutions that appear and disappear as the bond length varies. The gray curves denote the CAP-free solutions. Results from ref. 64 are marked as crosses.

The most striking finding is that the structure of CAP solutions is strongly dependent on the geometry. CAP calculations do not yield smooth potential energy curves, in contrast to what has been previously suggested.^39,51,64^ While each solution is itself continuous, they all vanish at some point. For both 0th- and 1st-order cases, the number of CAP solutions fluctuates between zero and four, which also contradicts the statement that the 1st-order results would be less prone to discontinuities.^64^

As the geometry changes, a minimum in the velocity profile can become progressively shallower, and if it coalesces with a local maxima, it vanishes. This happens in both directions of bond stretching. Moving away from the equilibrium distance (1.10 Å), solution a disappears at stretched (1.32 Å) but also at contracted (1.03 Å) bond lengths. While solution b vanishes upon less stretching (1.17 Å), it remains stable at shorter distances. We also find a solution, c, that is barely stable at the equilibrium geometry, visible in Fig. 7, and another solution, d, appearing at shorter distances. Even though they retain a localized π* character, solutions c and d have considerably larger *η*_opt_ and velocities than solutions a and b, thus being more perturbed by the CAP and less accurate. For the same reasons, the first-order corrected solutions a′ and b′ should be preferred over c′ and d′.

Although all CAP solutions have resonance positions and widths that decrease monotonically upon bond stretching, each pair of curves is remarkably different and far from parallel. Strictly imposing one of the criteria discussed in Sec. 3.1 would produce a unique but discontinuous potential energy curve, shifting from solutions b/b′ to a/a′ as the bond stretches. As shown in Fig. S9 of the SI, choosing the CAP solution with smallest *η*_opt_, discontinuities appear when it vanishes and the next smallest *η*_opt_ solution takes over. Taking the solution with the smallest velocity produces the same outcome, though with discontinuities at other geometries.

We come back to the smooth potential energy curves of ref. 64. In light of our results, inspection of their data reveals that solution a was patched with solution b between 1.05 Å and 1.10 Å (see Fig. 10), and similarly for solutions a′ and b′, between 1.20 Å and 1.25 Å (see Fig. 11). The position for the patching produces a potential energy curve that only appears smooth when visualized with a coarse grid. We also notice that, in contrast to what has been stated before,^64^ the CAP-free solutions are always continuous.

To preserve some degree of uniqueness and continuity of the potential energy curve, one is obliged to abandon one CAP solution as physical and pick a second one as more physical at particular geometries where the two pair of curves approximately cross (real and imaginary parts would hardly cross at the same point). However, there is nothing special at such geometries, as the CAP solutions can have very different values of *η*_opt_ and velocities, which is the case for N_2_^−^. Moreover, even if one chooses to alternate between CAP solutions, what would be the justification for discarding either of them at neighboring geometries to which they are adiabatically connected? Such inconsistencies only arise because one imposes a single solution as physical. Once again, a simpler alternative is to recognize that CAP calculations can yield various approximate solutions, and that the different and vanishing potential energy surfaces are a consequence of such non-uniqueness.

The results shown in Fig. 10 and 11 are obtained for a fixed CAP box, which does not adapt to the elongation of the bond. We further tested how the structure of CAP solutions changes by updating the onsets. We adopt the same procedure as in ref. 64, where each Cartesian onset is set to 〈*x*^2^〉^1/2^, where *x* is the electronic Cartesian coordinate and the expectation value is computed for the neutral wave function described at the CAP-free CCSD level of theory. In the SI, Table S8 compares the results obtained for fixed and flexible CAP onsets, at certain specific geometries, whereas Table S9 contains the onset values. We observe minute differences on the resonance parameters at stretched geometries, and somewhat larger differences at the contracted geometry. Importantly, the structure of CAP solutions does not change whether one chooses fixed or flexible onsets. However, we notice that the onsets themselves change little as a function of geometry. When the CAP onsets are allowed to change more, the impact on the solutions can be much more pronounced.^63^

Our potential energy curves for N_2_^−^ also reveal that CAP/EOM-EA-CCSD does not yields a consistent conversion between a resonance and a bound state, and that the degree of internal inconsistency is comparable to that of CAP/ΔUCCSD, in contrast to previous claims.^71^ These results are discussed in Sec. S2 of the SI, as they are not directly related to the multiple CAP solutions.

### 3.7 CAP calculations do not yield unique special points in potential energy surfaces

The appearance of multiple CAP solutions has important consequences when one tries to locate points of special significance in potential energy surfaces. To illustrate this point, we look once again into the potential energy curves of N_2_^−^ and the MEEP geometry of the chloroethylene anion.

Starting with the former, Fig. 12 shows that there is no unique equilibrium geometry of N_2_^−^ according to the CAP calculations. There is one in the 0th-order (a) and two in the 1st-order (a′ and b′) results. Not finding more than one 0th-order equilibrium geometry is fortuitous, as curve b would likely support an additional minimum by changing the CAP onset, for example. Other curves, not visible in the scale of the figure, disappear at even shorter distances.

**Fig. 12:**
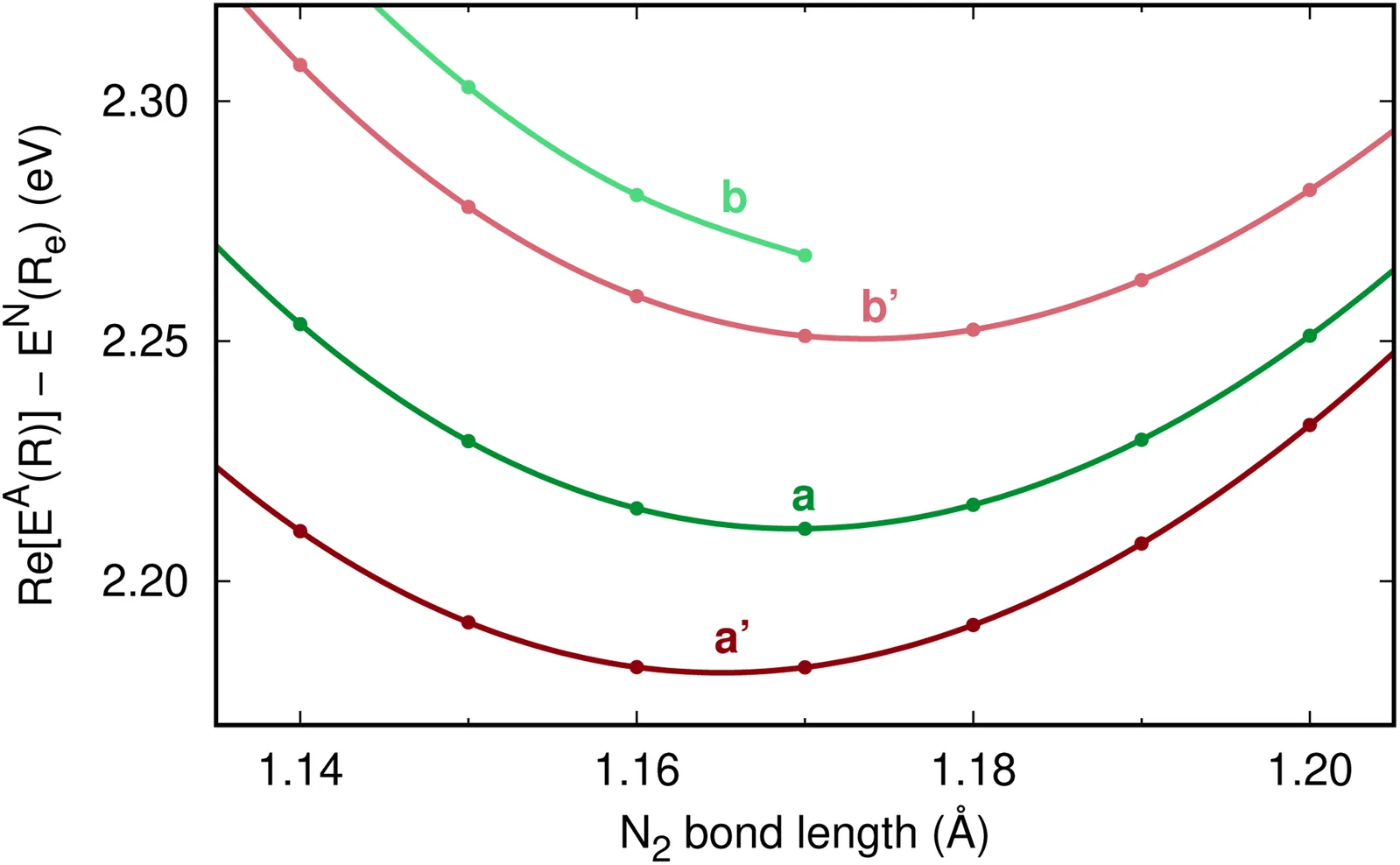
Real part of the potential energy curve of N_2_^−^, around its equilibrium structure, obtained with 0th-order (solutions a and b) and 1st-order (solutions a′ and b′) CAP/EOM-EA-CCSD/aug-cc-pVTZ+3s3p3d(C).

To gauge the impact of the non-unique geometries of N_2_^−^, we evaluate three spectroscopic parameters, namely, the equilibrium distance (*R*_0_), the harmonic vibrational frequency (*ω*), and the transition energy between the (harmonic) vibrational ground states of N_2_ and of N_2_^−^ (*E*_00_). These properties are obtained by considering only the real part of the resonance energy. The values of the three parameters are gathered in Table S10 of the SI.

To compare the errors stemming from the non-unique equilibrium geometry to those associated with the electronic structure method, Table S10 also includes available results obtained with three accurate methodologies,^53^ MRCI with singles and doubles plus Davidson correction [MRCISD+Q_P_], XMS-CASPT2, and EOM-EA-CCSD, all combined with the projected CAP formalism.^39^ Besides using projected CAP, the previous EOM-EA-CCSD result^53^ uses a slightly different basis set than ours. Results obtained without the Davidson correction and without the double excitations of MRCISD+Q_P_ show larger variations with respect to the three accurate methods mentioned above.^53^ A single set of parameters was reported for each method.^53^

These three methods, MRCISD+Q_P_, XMS-CASPT2, and EOM-EA-CCSD, rely on different approaches to describe electronic correlation, and yet all manage to deliver rather close spectroscopic parameters. The equilibrium distance *R*_0_ varies within 0.01 Å, the vibrational frequency *ω* shows fluctuations of around 200 cm^−1^, whereas the transition energy *E*_00_ oscillates within 0.10 eV. These ranges can thus be taken as reasonable estimates of the error associated with the electronic structure method.

Remarkably, the three CAP/EOM-EA-CCSD solutions yield similar variations, of 0.01 Å for *R*_0_, of around 100 cm^−1^ for *ω*, and of 0.07 eV for *E*_00_. Therefore, also for spectroscopic properties we find that the non-uniqueness error of CAP calculations are comparable to the error of accurate electronic structure methods.

To illustrate one last problem, we turn into the MEEP geometry of the chloroethylene anion, whose results are shown in Fig. 13. The SI contains a zoomed-out view (Fig. S10) and the numerical values (Table S11) of all solutions shown in Fig. 13. We notice that this geometry yields only approximate degenerate states, with an absolute energy difference (29 meV) that is double the one previously reported (14 meV).^44^

**Fig. 13:**
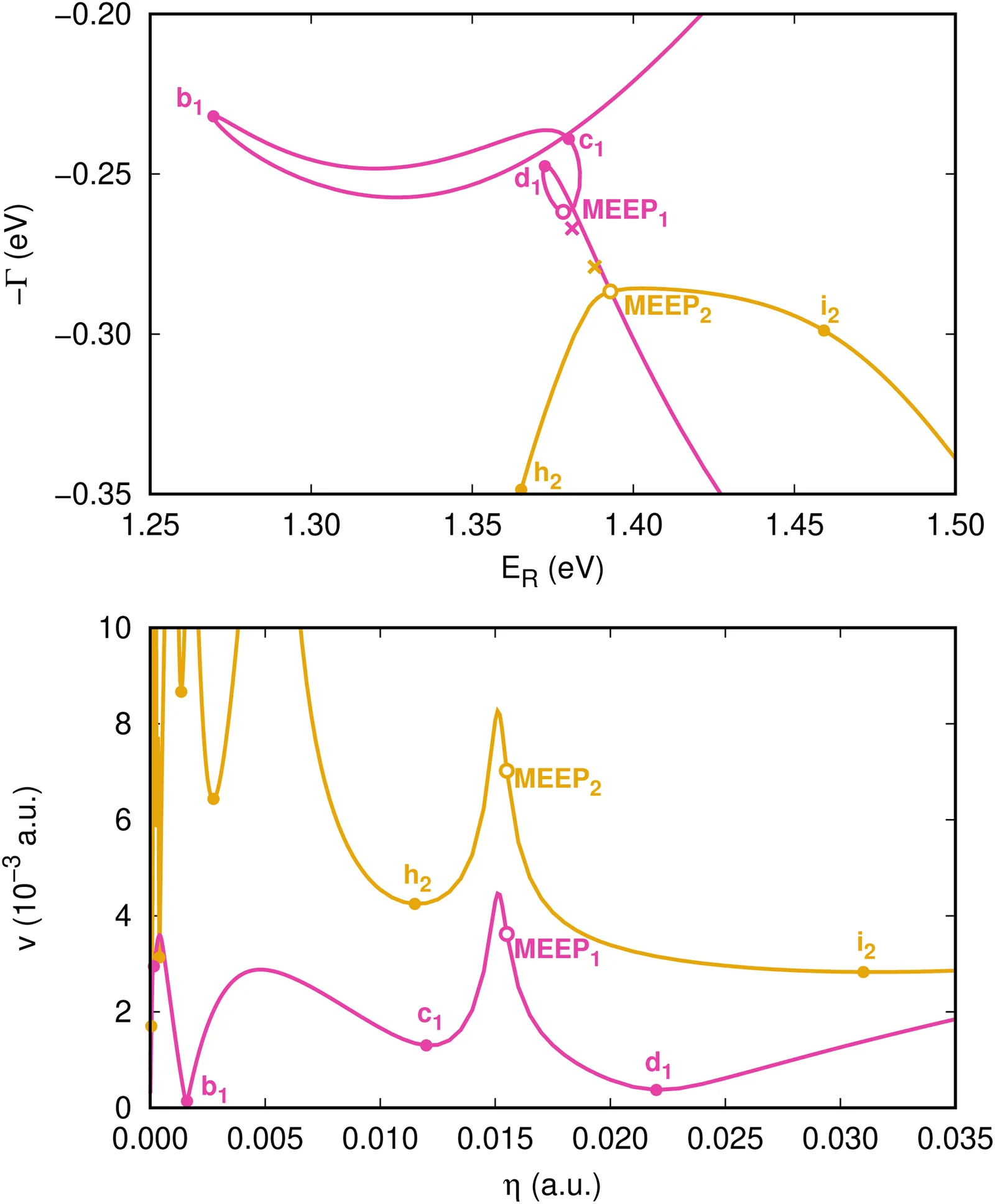
*η*-Trajectories (top) and velocities (bottom) of the chloroethylene anion at the MEEP geometry from ref. 44, optimized for *η* = 0.0155 a.u. The MEEP solutions are marked as open circles (present work) and crosses (ref. 44). Minima are marked as filled circles. Only the curves associated with the MEEP quasi-degeneracy are shown.

We find that the two states involved in the MEEP are not actual CAP solutions, but instead appear close to sharp maxima of the velocity. This is not coincidental. In this and other cases, we observe a fast increase in the velocities when two *η*-trajectories approach each other, reaching maximum values at their closest encounter. The *η*-trajectories behave as if they are strongly attracted to each other at short ranges. This behavior is probably rooted in the self-orthogonality of the two states at the exceptional point.^20^ From the inverse correlation between maximum velocities and shortest distances, we suspect that the velocities diverge at degeneracy. That being the case, the states responsible for the MEEP geometry would never be a standard CAP solution (a local minima of the velocity), a crucial limitation of the velocity optimization criterion (and also of the *ξ* function).

Even though the MEEP solutions are not CAP solutions, the latter exist at other values of *η*_opt_. Each *η*-trajectory supports one solution to each side of the MEEP maxima, whereas another low-velocity solution (b_1_) can also be found (see Fig. 13). The associated Dyson orbitals can be seen in Fig. 14. The low-*η*_opt_ solutions have well defined π* and σ* characters, whereas the MEEP solutions and the high-*η*_opt_ solutions acquire a mixed π*/σ* character. All solutions span approximately 0.2 eV in the resonance position and 0.1 eV in the resonance width, much less than for the equilibrium geometry.

**Fig. 14:**
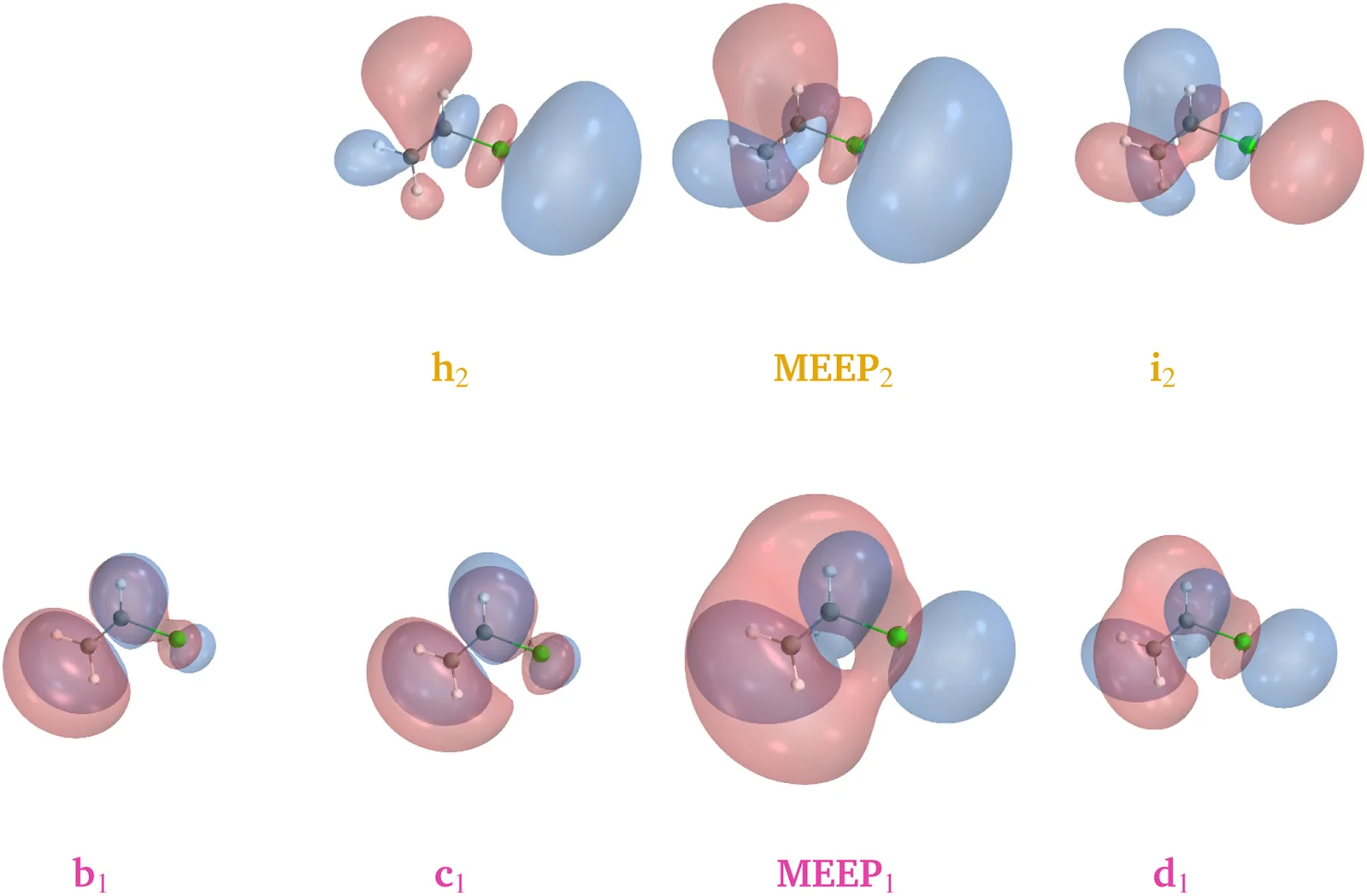
Real part of the Dyson orbital for the selected points shown in Fig. 13, for an isovalue of 0.02.

Finally, we notice that this MEEP geometry has been obtained at a fixed *η*, by optimizing a particular state describing the π* resonance until it became almost degenerate with a particular state having σ* character.^44^ By picking a different *η*, a distinct MEEP geometry would have been found. Although we did not do this here, a significant impact on the MEEP energies can be expected based on the set of results discussed above. Dissociative processes mediated by resonances are highly sensitive to the landscape of the potential energy surfaces and their critical points, specially when short-lived σ* resonances are involved. To gain insights about the dissociation mechanisms based on the MEEP geometry and energy, it needs to be accurately and uniquely identified, which current CAP calculations struggle to do.

## 4 Conclusions

We have presented an extensive investigation on the appearance of multiple solutions in CAP calculations for anionic resonances. We argue that the standard practice of selecting one solution as “physical” is not justifiable in general. By lifting the assumption of a single solution, we acknowledge them as different ways in which the resonance can be stabilized by the CAP in finite basis set calculations.

The occurrence of different CAP solutions is more pervasive and has more serious consequences than commonly assumed. They represent a large source of uncertainty in the calculation of resonance energies and properties, which we found to be comparable to and often larger than the errors associated with the basis set incompleteness and the approximate electronic structure method.

We demonstrated that such non-uniqueness cannot be eliminated and not even mitigated with any of the strategies that have been proposed so far. While the number of CAP solutions is relatively independent on the basis set, persisting for very large bases, it actually increases when removing the first-order energy contribution stemming from the CAP. In turn, adding a real perturbation to the CAP destabilizes all solutions in a similar way, regardless of their character.

We further explored how the structure of CAP solutions is affected by different methodological choices. We find comparatively small effects associated with the description of electronic correlation, the shape of the CAP, and whether the velocity or the *ξ* error function is optimized. In contrast, the basis set has a dramatic and non-trivial effect. Taken together with the findings of ref. 63, we conclude that the basis set and the CAP onsets are the key aspects behind the emergence of several solutions.

Their consequences are even more daring for potential energy surfaces, with solutions appearing and vanishing in both 0th- and 1st-order results. Moreover, the equilibrium structures of resonances and exceptional points are not uniquely defined with current CAP calculations, which has grave implications for modeling electron-induced reactions.

Describing resonances with the CAP method is still far from being as black-box as describing bound states with standard quantum chemistry methods. The dubious assignment of resonant states and the large non-uniqueness error we have brought to light, in addition to the arbitrary choice of CAP onsets, are the main obstacles for the methodology. We recommend that future investigations relying on the CAP method mention whether more than one solution is found, supported by the presentation of *η*-trajectories and velocities. Assignments of one or more CAP solutions to resonant states should be properly justified, with the associated non-uniqueness error estimated. Here, we have proposed a simple way to identify admissible CAP solutions and to quantify the uncertainty associated with their non-uniqueness. While alternative protocols can be developed in the future, a persistent challenge lies on the arbitrariness in separating admissible and non-admissible CAP solutions. However, this does not change our main conclusion that the appearance of multiple solutions can introduce large uncertainties in CAP calculations.

The issue is not exclusive of the CAP method, and may also affect other non-Hermitian methodologies such as complex scaling^10,11^ and the method of complex basis functions.^13,14^ Although the problem has been previously recognized,^14,73,74^ to the best of our knowledge only one application has quantified it: for the lowest anionic resonance of s-triazine, two solutions appear separated by around 0.02 eV.^14^ While this is much less than what we have observed with the CAP method, other studies do not mention multiple solutions. The extent of the issue in other non-Hermitian methods thus remains largely unexplored. Stabilization methods^8,9^ offer another approach to describe resonances, by relying, for instance, on Padé approximants to perform an analytic continuation of the energy into the complex plane.^75^ In addition to supporting multiple solutions, the details on the analytic continuation introduce further uncertainties to the determination of resonance energies. Such uncertainties have been more extensively documented,^76,77^ and appear to be smaller than the non-uniqueness error of CAP calculations. Assessing such non-uniqueness errors across different methodologies is an interesting direction to be explored.

Why does the CAP method yield multiple solutions? That is the key question arising from the present contribution and that we hope to address in the future.

## Author contributions

Fábris Kossoski: conceptualization, formal analysis, funding acquisition, investigation, methodology, validation, visualization, writing – original draft, writing – review & editing.

## Conflicts of interest

There are no conflicts to declare.

## Supplementary Material

SC-017-D6SC03887E-s001

## Data Availability

The data supporting this article have been included as part of the supplementary information (SI). Supplementary information is available. See DOI: https://doi.org/10.1039/d6sc03887e.
