# Supplementary material for: On the multiple solutions of the complex absorbing potential method for resonances
Source: Chem Sci. 2026 Sep 4;17(43):21064–80. doi: 10.1039/d6sc03887e (PMC13618336; doi:10.1039/d6sc03887e)
Supplement: Supplementary file 1 [file SC-017-D6SC03887E-s001.pdf]

Supplementary Information (SI) for Chemical Science.
This journal is © The Royal Society of Chemistry 2026

Supporting Information for "On the multiple solutions of the complex absorbing potential method for resonances"

Authors[1, a)]

Affiliation

a)Electronic mail: email

S1. GENERATION OF η-TRAJECTORIES

To describe resonant states with CAP calculations, a sufficiently large number of states have to be calculated. Because complex-valued energies cannot be ordered, and because the set of converged states is not the same across different regimes of η, the number of states required is not known beforehand. For very small η, when all states remain close to the real axis, we calculate as many states as needed such that the highest-lying real energy is safely (> 1 eV) above the expected position of the resonance. As η increases, most states acquire large imaginary energies (as they describe continuum-like states), whereas one or few retain a relatively small imaginary component (indicative of a resonant-like state). Therefore, we carefully but progressively decrease the number of calculated states as η increases, which is also needed in many cases to reach convergence.

From the collection of complex-valued energies $E^{(i)}(\eta_p)$, where (i) denotes the index of the state, we had to generate a series of η-trajectories. To find the best pairing between the set of states obtained at two sequential points, η_p and η_{p+1}, we compare the calculated energies at η_{p+1} with the estimated values obtained by linearly extrapolating the energies at η_p using their derivatives. For each pair of states (i, j), with i evaluated at η_p and j at η_{p+1}, the absolute difference between the calculated and the estimated energies is defined as

$$\Delta E^{(i,j)} = \left| E^{(j)}(\eta_{p+1}) - E^{(i)}(\eta_p) - \frac{dE^{(i)}}{d\eta}(\eta_p)(\eta_{p+1} - \eta_p) \right| \tag{S1}$$

In the first iteration, the pair with the smallest $\Delta E^{(i,j)}$ is connected as a single η-trajectory. After excluding these two states, the next smallest $\Delta E^{(i,j)}$ produces the second η-trajectory, and so on. The procedure stops when the smallest allowed $\Delta E^{(i,j)}$ lies above a given threshold. We fix this threshold at 10^{-4} E_h, which is small enough to guarantee that most points are correctly assembled into smooth η-trajectories, yet sufficiently large to avoid the incorrect merging of separate η-trajectories.

S2. CAP CALCULATIONS DO NOT YIELD INTERNALLY CONSISTENT POTENTIAL ENERGY SURFACES

The exact resonance width becomes zero at the same geometry where the resonance position goes to zero. A method that fulfills this condition is said to be internally consistent.

It has been claimed that the 1st-order CAP/EOM-EA-CCSD method would yield internally consistent potential energy surfaces, whereas the 0th-order variant would not.[1] It has also been stated that CAP/ΔUCCSD would be much less internally consistent than CAP/EOM-EA-CCSD.[2] Here, by performing the same calculations as in these previous studies,[1,2] but with a fine grid of points (0.01 Å), we demonstrate that neither of these two statements is correct.

Fig. S1 shows our 1st-order CAP/EOM-EA-CCSD results for N_2^-, the same as in Fig. 11 of the manuscript, but now focusing on the internuclear region where the resonance becomes a bound state. We find that Γ is non-negligible (13 meV) at the crossing ($E_R = 0$), which occurs at around 1.41 Å. Beyond the crossing, the anion would be bound, as $E_R < 0$, whereas a positive imaginary part, $\Gamma > 0$, suggests that it would still be unbound. In other words, 1st-order CAP/EOM-EA-CCSD is not internally consistent. A positive value of Γ extends for another 0.05 Å, after which the CAP solution vanishes.

The CAP-free solutions are also shown in Fig. S1. At 1.45 Å and 1.50 Å, they coincide with the results labeled as 1st-order CAP/EOM-EA-CCSD in Ref. 1, and despite the CAP calculation still yielding one CAP solution at 1.45 Å. Patching the curves obtained with and without the CAP merely imposes a discontinuous conversion between resonance and bound state. This does not mean that the electronic structure method used in combination with the CAP is internally consistent. In fact, Fig. S1 shows that the resonance width never reaches zero, even past the crossing point. This is not too surprising. Although the anion is stable at this region, the CAP still inflicts an artificial imaginary part to the energy, just as what is observed for the neutral system.

Fig. S1 also compares our CAP/EOM-EA-CCSD and CAP/ΔUCCSD results, both calculated with the 1st-order correction. CAP/ΔUCCSD yields a slightly smaller value of Γ at the crossing (10 meV) when compared to CAP/EOM-EA-CCSD (13 meV). The degree of internal (in)consistency is thus similar, with a slight edge for the state-specific calculation. The fact that EOM-EA yields anion and neutral states from the same similarity-transformed Hamiltonian, while state-specific CC relies on separate calculations, argued in Ref. 2 to explain a supposed advantage of the former, seems to be unimportant as far as the conversion between resonance and bound state

is concerned.

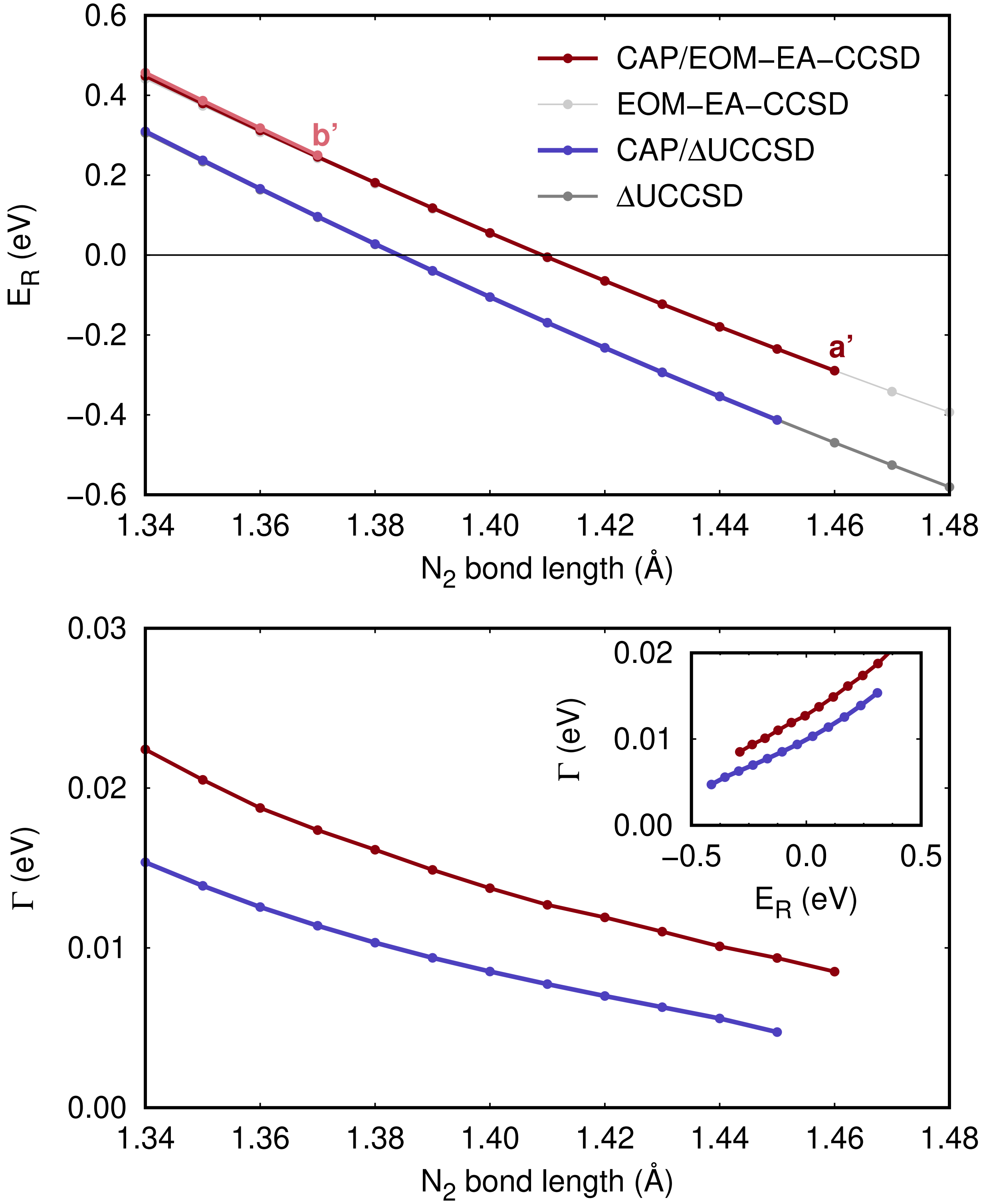

FIG. S1. Close-up of the resonance position (E_R) and width (Γ) of $N_2{}^-$, obtained with 1st-order CAP/EOM-EA-CCSD, CAP/ΔUCCSD, and their CAP-free analogues, with the aug-cc-pVTZ+3s3p3d(C) basis set. The inset displays Γ as a function of E_R.

Equilibrium geometry of N_2, in atomic units, taken from Ref. 3:

```
N        0.0000000000     0.0000000000    -1.0370000000
N        0.0000000000     0.0000000000     1.0370000000
```

Equilibrium geometry of chloroethylene, in Angstrom, taken from Ref. 4:

```
Cl       1.0680104854    -0.1012164653     0.0000000000
C       -0.5726172716     0.5120761592     0.0000000000
C       -1.6486244026    -0.2890338484     0.0000000000
H       -0.6214425547     1.6022133087     0.0000000000
H       -1.5614635547    -1.3774595164     0.0000000000
H       -2.6458220969     0.1576722528     0.0000000000
```

Geometry of the chloroethylene anion at its minimum energy exceptional point, in Angstrom, taken from Ref. 4:

```
Cl       1.1442281526    -0.1042390892    -0.0015428154
C       -0.6746057807     0.5748499999     0.0119123793
C       -1.7305633941    -0.3261625210    -0.0041175910
H       -0.6977644357     1.6689035631    -0.0188162823
H       -1.5502833544    -1.4061632188     0.0143651081
H       -2.7728157558     0.0171992993    -0.0160896945
```

TABLE S1. CAP onsets (in a_0) for N_2, taken from Ref. 3, and for chloroethylene, at its neutral equilibrium geometry (eq.) and at the minimum energy exceptional point (MEEP) of the anion, taken from Ref. 4.

System	x_0	y_0	z_0
N_2	2.760	2.760	4.880
chloroethylene (eq.)	13.690	6.460	4.545
chloroethylene (MEEP)	14.445	6.670	4.585

TABLE S2. Resonance position (E_R), resonance width (Γ), optimized CAP strength ($\eta_{\rm opt}$), velocity [$v(\eta_{\rm opt})$], and $\Delta r = \langle r^2 \rangle_A^{1/2} - \langle r^2 \rangle_N^{1/2}$, where A/N indicates that the expected value is evaluated for the anion/neutral wave function, for selected CAP solutions of the A'' and A' symmetries of the chloroethylene anion, at the neutral equilibrium geometry, calculated with 0th-order CAP/EOM-EA-CCSD/aug-cc-pVDZ+3p(A). The associated η-trajectories and velocities are shown in Fig. 3 of the manuscript.

Symmetry	Solution	E_R (eV)	Γ (eV)	$\eta_{\rm opt}$ (10^{-5} a.u.)	$v(\eta_{\rm opt})$ (10^{-3} a.u.)	Δr (Å)
A''	a	1.631	0.532	196	0.68	0.96
	b	1.804	0.261	1720	0.99	0.77
	c	2.405	0.401	136000	2.08	0.60
A'	e_1	1.212	0.963	258	6.75	2.02
	f_1	1.731	1.294	1120	14.95	1.75
	g_1	2.272	1.115	4070	4.40	1.44
	h_1	2.893	1.263	30500	8.17	1.00
	i_1	3.865	0.943	355000	5.71	0.58
	c_2	2.062	1.174	475	1.90	2.10
	d_2	2.134	1.177	755	6.76	1.76
	e_2	2.404	1.263	1875	8.90	1.41
	f_2	2.707	1.457	3850	13.07	1.53
	g_2	3.250	1.696	11400	8.86	0.79
	h_2	3.448	2.466	23100	15.61	0.71
	i_2	3.993	1.806	70500	2.73	0.97
	a_3	3.356	0.844	460	4.08	1.51
	b_3	3.300	1.341	1620	2.88	1.44

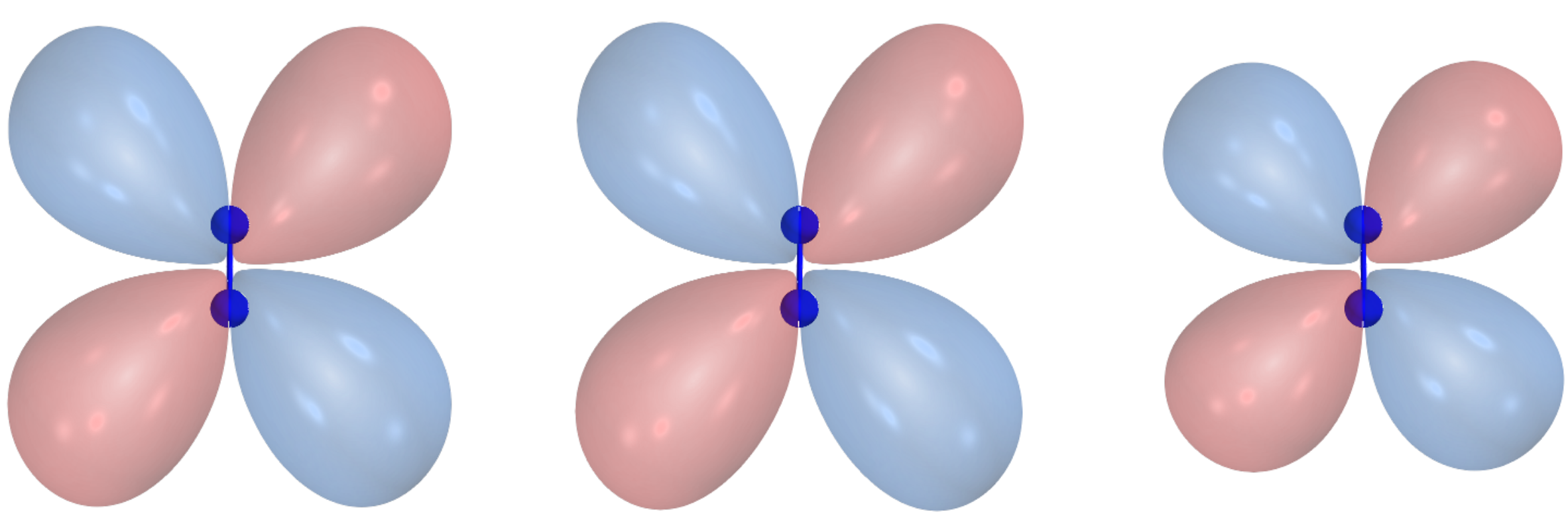

(a) (b) (c)

FIG. S2. Real part of the Dyson orbital for the three 0th-order solutions obtained with CAP/EOM-EA-CCSD/aug-cc-pV5Z+3s3p3d(A), for an isovalue of 0.02. The associated η-trajectory and velocity are shown in Fig. 6 of the manuscript.

TABLE S3. Resonance position (E_R), resonance width (Γ), optimized CAP strength (η_{opt}), and its associated velocity [$v(\eta_{\text{opt}})$], for solutions b and g_1 of the π^* and σ^* shape resonances of the chloroethylene anion, at the neutral equilibrium geometry, along with the values reported in previous studies.

State	Method	E_R (eV)	Γ (eV)	η_{opt} (10^{-5} a.u.)	$v(\eta_{\text{opt}})$ (10^{-3} a.u.)
π^*	CAP/EOM-EA-CCSD (b)[a]	1.804	0.261	1720	0.99
	CAP/EOM-EA-CCSD[b]	1.801	0.260	1650	-
	CAP/EOM-EA-CCSD[c]	1.730	0.266	1000	-
	CAP/ADC(2)[d]	1.59	0.42	-	-
	CAP/SAC-CI[e]	1.718	0.312	-	-
	CAP/XMS-CASPT2[e]	1.810	0.321	-	-
	electron transmission experiment[f]	1.28	-	-	-
σ^*	CAP/EOM-EA-CCSD (g_1)[a]	2.272	1.115	4070	4.40
	CAP/EOM-EA-CCSD[b]	2.268	1.115	4000	-
	CAP/ADC(2)[d]	2.36	0.97	-	-
	CAP/SAC-CI[e]	2.530	0.817	-	-
	CAP/XMS-CASPT2[e]	2.65	-	-	-
	electron transmission experiment[f]	2.84	-	-	-

[a] aug-cc-pVDZ+3p(A), present work.
[b] aug-cc-pVDZ+3p(A), Refs. 4 and 5.
[c] aug-cc-pVTZ+3p(A), Ref. 5.
[d] cc-pVDZ+1s8p2d1f(A), Ref. 6.
[e] cc-pVDZ+2s5p2d(A), Ref. 7.
[f] Refs. 8 and 9. Experimental uncertainties of 0.03 eV.

TABLE S4. Resonance position (E_R), resonance width (Γ), optimized CAP strength (η_{opt}), and its associated velocity [$v(\eta_{opt})$], for N_2^- at the neutral equilibrium geometry, calculated with 0th-order (solutions *a* and *b*) and 1st-order (solutions *a′* and *b′*) CAP/EOM-EA-CCSD/aug-cc-pVTZ+3s3p3d(C). The associated 0th-order η-trajectories and velocities are shown in Fig. 5 of the manuscript.

Solution	Method	E_R (eV)	Γ (eV)	η_{opt} (10^{-5} a.u.)	$v(\eta_{opt})$ (10^{-3} a.u.)
a	CAP/ΔUHF[a]	2.798	0.164	111	0.06
	CAP/EOM-EA-CCSD[a]	2.488	0.425	157	1.98
	CAP/ΔUCCSD[a]	2.451	0.361	150	1.60
	CAP/MRCIS[d]	2.849	0.680	162	4.36
	CAP/qsGW[e]	2.565	0.460	160	1.71
	CAP/EOM-EA-CCSD[b]	2.487	0.417	150	-
	CAP/FCI[f]	2.449	0.391	150[h]	-
b	CAP/EOM-EA-CCSD[a]	2.618	0.395	710	2.36
	CAP/ΔUCCSD[a]	2.559	0.359	660	2.29
	CAP/MRCIS[d]	3.062	0.403	948	1.42
	CAP/qsGW[e]	2.707	0.386	780	1.68
	CAP/EOM-EA-CCSD[c]	2.617	0.395	700	-
a′	CAP/ΔUHF[a]	2.778	0.150	235	0.19
	CAP/EOM-EA-CCSD[a]	2.412	0.469	262	4.94
	CAP/ΔUCCSD[a]	2.391	0.390	260	3.46
b′	CAP/ΔUHF[a]	2.827	0.129	1340	0.19
	CAP/EOM-EA-CCSD[a]	2.571	0.278	1430	0.32
	CAP/ΔUCCSD[a]	2.519	0.239	1420	0.27
	CAP/MRCIS[d]	3.00	0.31	1800	-
	CAP/EOM-EA-CCSD[b]	2.571	0.255	1190/1480[i]	-
	CAP/EOM-EA-CCSD[c]	2.572	0.279	1750	-
	Experiment[g]	2.316	0.414	-	-

[a] Present work.
[b] Ref. 3.
[c] Ref. 10.
[d] Ref. 11.
[e] Ref. 12.
[f] Ref. 13.
[g] Ref. 14.
[h] Evaluated at the estimated optimal η reported in Ref. 3 for CAP/EOM-EA-CCSD.
[i] Optimized separately for resonance position and width.

TABLE S5. Resonance position (E_R), resonance width (Γ), optimized CAP strength (η_{opt}), and its associated velocity [$v(\eta_{\text{opt}})$], for $N_2{}^-$ at the neutral equilibrium geometry, calculated with 0th-order CAP/EOM-EA-CCSD and various basis sets. We report values for all the CAP solutions with $\eta_{\text{opt}} < 0.2$ a.u. The associated η-trajectories and velocities are shown in Fig. 6 of the manuscript.

Basis set	Solution	E_R (eV)	Γ (eV)	η_{opt} (10^{-5} a.u.)	$v(\eta_{\text{opt}})$ (10^{-3} a.u.)
aug-cc-pVDZ+3s3p3d(C)	*a*	2.778	0.380	545	0.18
	b	3.157	0.986	5775	10.36
aug-cc-pVTZ+3s3p3d(C)	*a*	2.488	0.425	157	1.98
	b	2.618	0.395	710	2.36
	c	3.258	1.371	18000	14.85
aug-cc-pVQZ+3s3p3d(C)	*a*	2.509	0.370	380	1.40
	b	2.657	0.508	1760	5.22
aug-cc-pV5Z+3s3p3d(C)	*a*	2.457	0.383	249	0.98
	b	2.595	0.434	1200	3.75
aug-cc-pVDZ+3s3p3d(A)	*a*	2.588	0.670	132	4.40
	b	2.802	0.449	735	1.79
aug-cc-pVTZ+3s3p3d(A)	*a*	2.526	0.502	138	1.82
	b	2.556	0.406	372	2.17
aug-cc-pVQZ+3s3p3d(A)	*a*	2.472	0.520	76	0.29
	b	2.461	0.435	199	1.74
	c	2.581	0.410	890	3.20
aug-cc-pV5Z+3s3p3d(A)	*a*	2.461	0.542	61	1.38
	b	2.432	0.479	142	1.62
	c	2.542	0.389	720	2.84
aug-cc-pVTZ+6s6p6d(A)	*a*	2.572	0.568	62	1.94
	b	2.529	0.516	140	2.26
	c	2.565	0.404	410	2.21
aug-cc-pVTZ+6s6p6d6f(A)	*a*	2.562	0.598	67	2.06
	b	2.520	0.482	188	2.05
	c	2.595	0.412	590	2.90

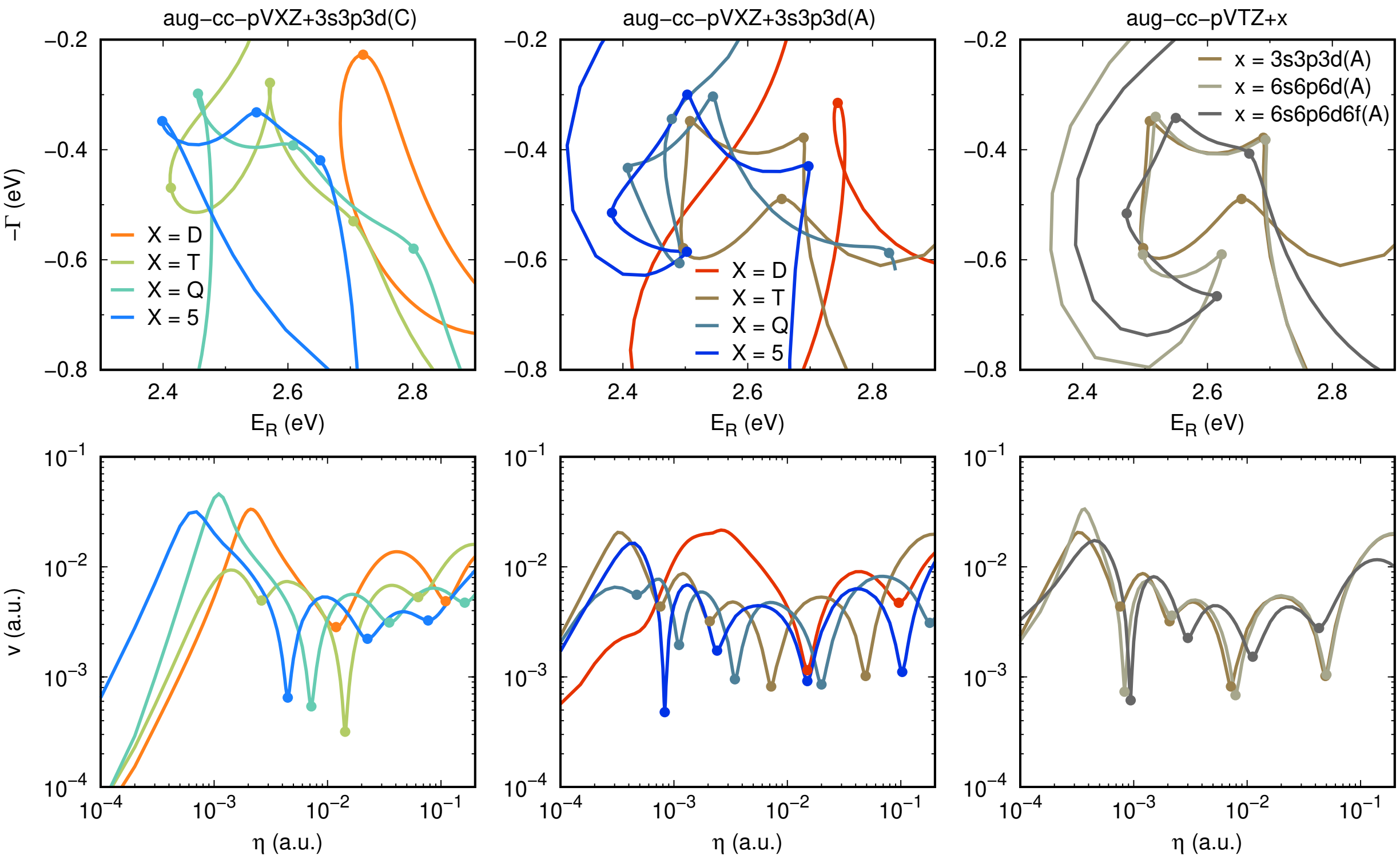

FIG. S3. η-trajectories (top) and velocities (bottom) for $N_2{}^-$, calculated with 1st-order CAP/EOM-EA-CCSD and various basis sets. Minima are marked as circles. For a variable valence set (X) increasing from double-zeta to quintuple-zeta, the extra set of 3s3p3d functions is placed either at the geometric center of the molecule (left column) or at each atom (middle column), or, on top of the aug-cc-pVTZ basis set, the extra set of functions (x) increases from 3s3p3d(A) to 6s6p6d6f(A) (right column).

TABLE S6. Resonance position (E_R), resonance width (Γ), optimized CAP strength (η_{opt}), and its associated velocity [$v(\eta_{\text{opt}})$], for N_2^- at the neutral equilibrium geometry, calculated with 1st-order CAP/EOM-EA-CCSD and various basis sets. We report values for all the CAP solutions with $\eta_{\text{opt}} < 0.2$ a.u. The associated η-trajectories and velocities are shown in Fig. S3.

Basis set	Solution	E_R (eV)	Γ (eV)	η_{opt} (10^{-5} a.u.)	$v(\eta_{\text{opt}})$ (10^{-3} a.u.)
aug-cc-pVDZ+3s3p3d(C)	a'	2.721	0.227	1190	2.84
	b'	3.043	0.731	11000	4.87
aug-cc-pVTZ+3s3p3d(C)	a'	2.412	0.469	262	4.94
	b'	2.571	0.278	1430	0.32
	c'	2.705	0.530	6300	5.31
aug-cc-pVQZ+3s3p3d(C)	a'	2.456	0.298	720	0.54
	b'	2.608	0.392	3500	3.12
	c'	2.801	0.580	16200	4.73
aug-cc-pV5Z+3s3p3d(C)	a'	2.399	0.348	446	0.65
	b'	2.550	0.332	2250	2.22
	c'	2.652	0.419	7700	3.26
aug-cc-pVDZ+3s3p3d(A)	a'	2.744	0.315	150	1.15
	b'	2.977	0.637	960	4.72
aug-cc-pVTZ+3s3p3d(A)	a'	2.655	0.490	76	4.36
	b'	2.497	0.579	207	3.20
	c'	2.508	0.348	720	0.82
	d'	2.690	0.378	490	1.02
aug-cc-pVQZ+3s3p3d(A)	a'	2.478	0.344	47	5.57
	b'	2.491	0.606	111	1.95
	c'	2.408	0.433	345	0.95
	d'	2.544	0.303	2000	0.85
aug-cc-pV5Z+3s3p3d(A)	a'	2.503	0.585	83	0.48
	b'	2.382	0.515	240	1.73
	c'	2.503	0.300	1500	0.92
	d'	2.697	0.430	10300	1.11
aug-cc-pVTZ+6s6p6d(A)	a'	2.623	0.590	83	0.73
	b'	2.497	0.591	217	3.61
	c'	2.517	0.340	790	0.68
	d'	2.693	0.382	5000	1.05
aug-cc-pVTZ+6s6p6d6f(A)	a'	2.615	0.666	94	0.61
	b'	2.471	0.516	300	2.26
	c'	2.549	0.342	1120	1.53
	d'	2.667	0.407	4300	2.78

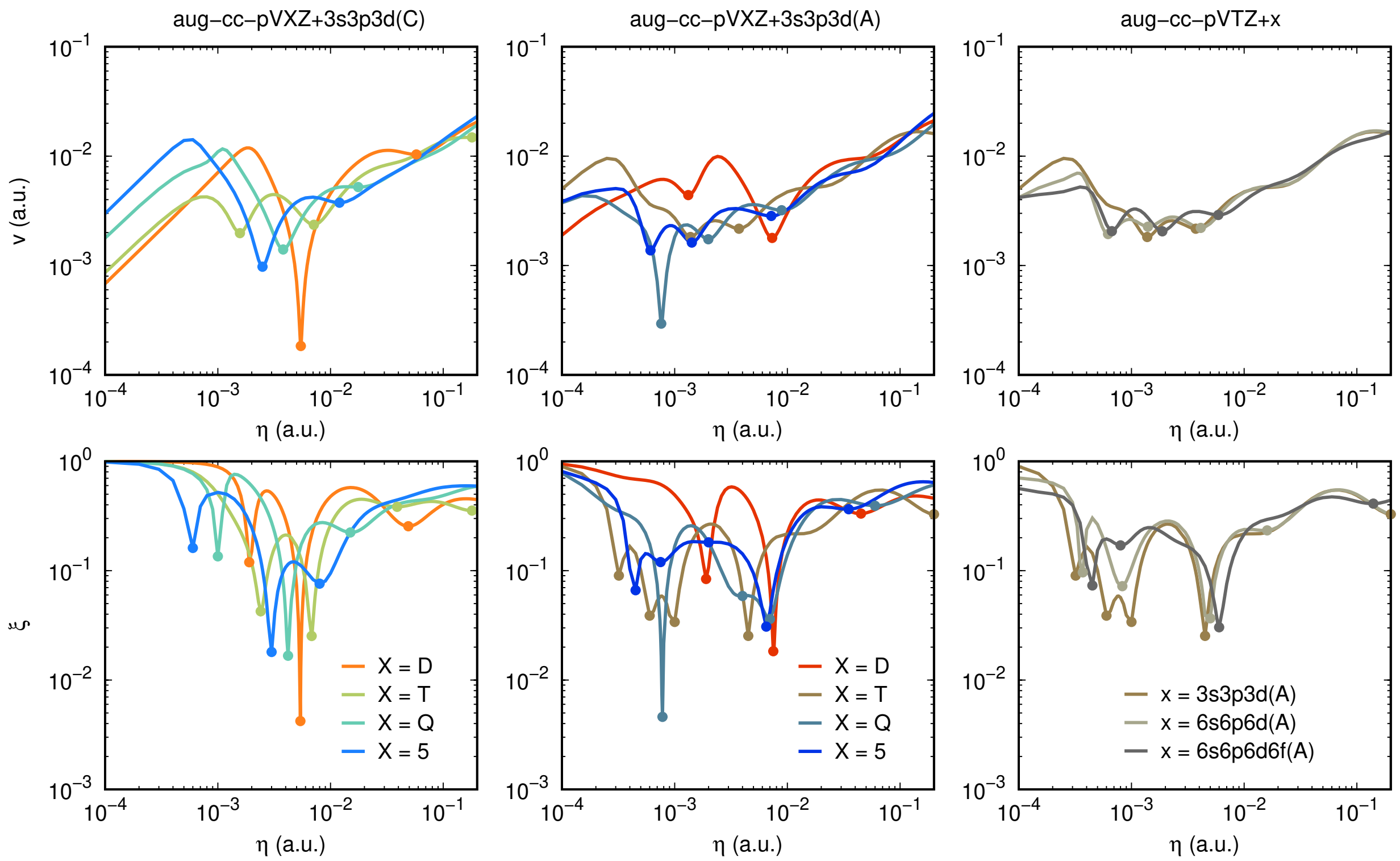

FIG. S4. The velocity (top) and the ξ function (bottom), defined by eqn (3) and eqn (9) of the manuscript, for $N_2{}^-$, obtained with 0th-order CAP/EOM-EA-CCSD and various basis sets. The corresponding η-trajectories are shown in Fig. 6 of the manuscript. Minima are marked as circles. For a variable valence set (X) increasing from double-zeta to quintuple-zeta, the extra set of 3s3p3d functions is placed either at the geometric center of the molecule (left column) or at each atom (middle column), or, on top of the aug-cc-pVTZ basis set, the extra set of functions (x) increases from 3s3p3d(A) to 6s6p6d6f(A) (right column).

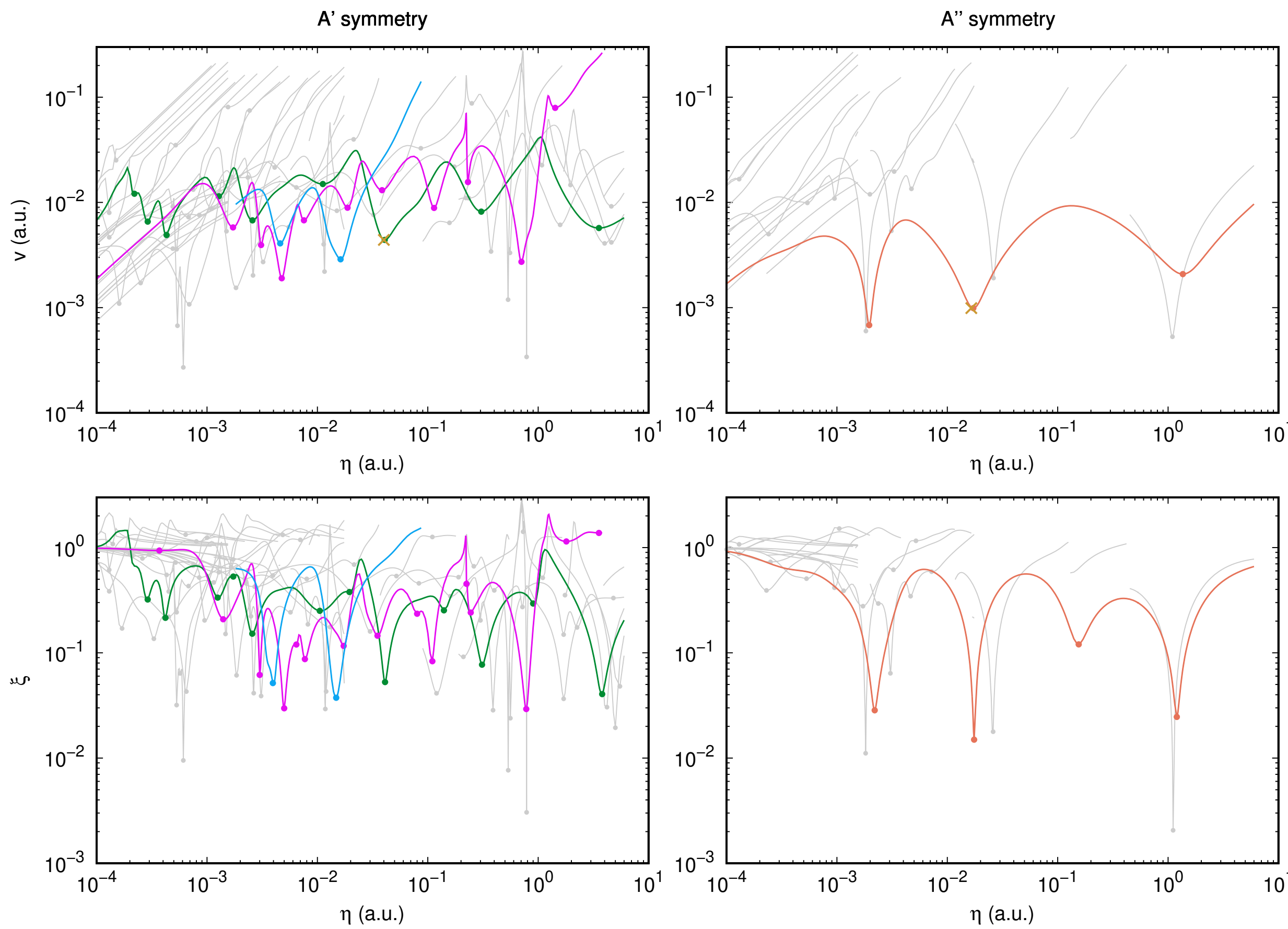

FIG. S5. The velocity (top) and the ξ function (bottom), defined by eqn (3) and eqn (9) of the manuscript, obtained for the A′ (left) and A′′ (right) symmetries of the chloroethylene anion, with 0th-order CAP/EOM-EA-CCSD/aug-cc-pVDZ+3p(A). The corresponding η-trajectories are shown in Fig. 3 of the manuscript. Those supporting solutions with localized σ^* (A′) and π^* (A′′) characters are highlighted. Minima are marked as circles. Results from Ref. 4 are marked as crosses.

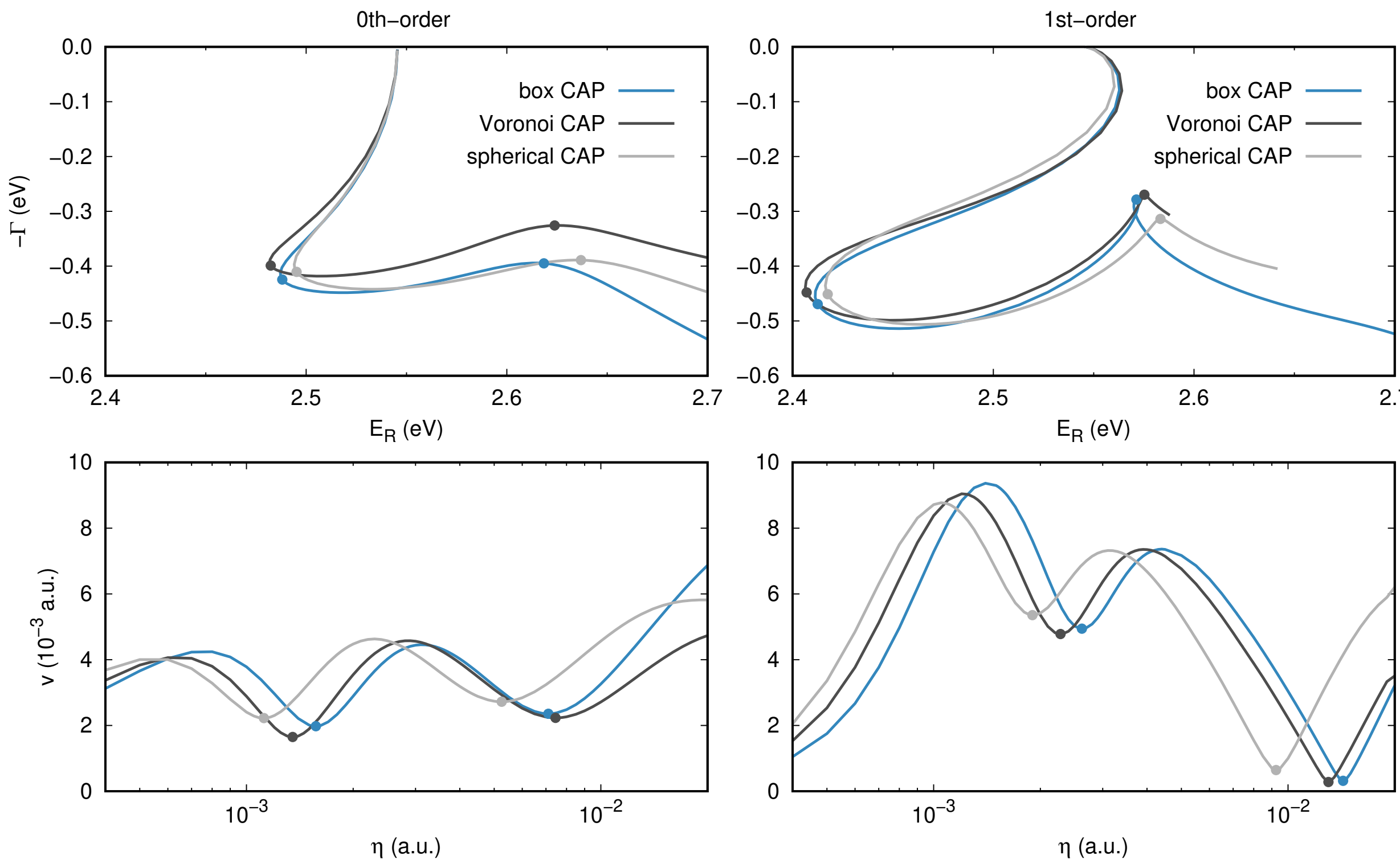

FIG. S6. η-trajectories (top) and velocities (bottom) for $N_2{}^-$, calculated with 0th-order (left) and 1st-order (right) CAP/EOM-EA-CCSD/aug-cc-pVTZ+3s3p3d(C), for box, spherical, and smoothed Voronoi CAPs. The onset for the latter two is equal to $4\,a_0$, whereas those for the box are given in Table S1. The values at the minima, marked by circles, and presented in Table S7.

TABLE S7. Resonance position (E_R), resonance width (Γ), optimized CAP strength (η_{opt}), and its associated velocity [$v(\eta_{\text{opt}})$], for the 0th-order (a and b) and 1st-oder (a' and b') solutions of the η-trajectories shown in Fig. S6.

Solution	CAP shape	E_R (eV)	Γ (eV)	η_{opt} (10^{-5} a.u.)	$v(\eta_{\text{opt}})$ (10^{-3} a.u.)
a	box	2.488	0.425	157	1.98
	Voronoi	2.482	0.399	135	1.65
	spherical	2.495	0.410	112	2.22
b	box	2.618	0.395	710	2.36
	Voronoi	2.624	0.326	745	2.23
	spherical	2.637	0.389	525	2.72
a'	box	2.412	0.469	262	4.94
	Voronoi	2.407	0.448	228	4.78
	spherical	2.417	0.451	190	5.36
b'	box	2.571	0.278	1430	0.32
	Voronoi	2.575	0.270	1300	0.28
	spherical	2.583	0.314	925	0.64

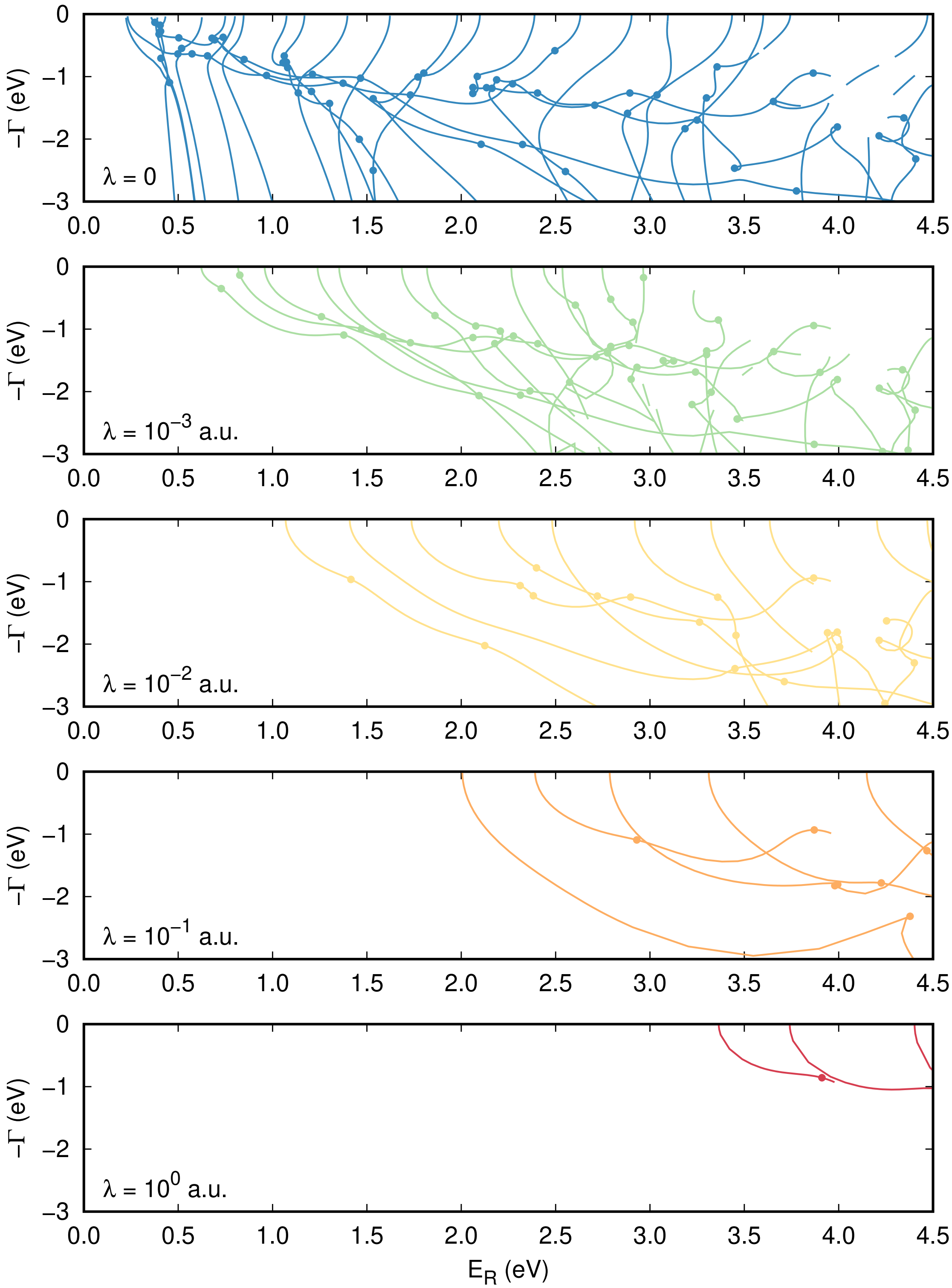

FIG. S7. η-trajectories for the A′ symmetry of the chloroethylene anion, calculated with 0th-order CAP/EOM-EA-CCSD/aug-cc-pVDZ+3p(A), for five different values of λ, defined in eqn (10) of the manuscript. Minima are marked as circles. Results for $\lambda = 0$ are the same as those shown in Fig. 3 of the manuscript.

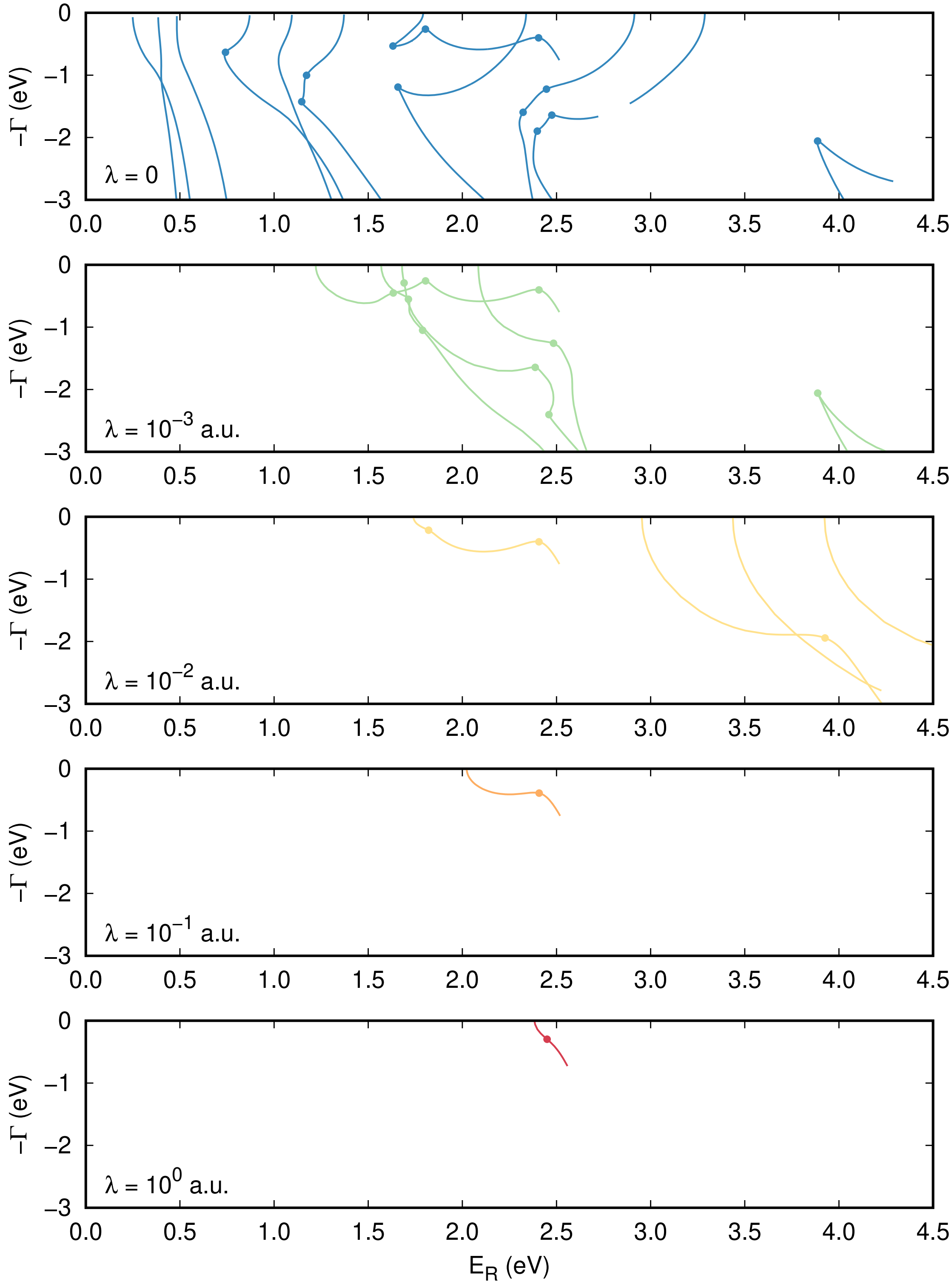

FIG. S8. η-trajectories for the A″ symmetry of the chloroethylene anion, calculated with 0th-order CAP/EOM-EA-CCSD/aug-cc-pVDZ+3p(A), for five different values of λ, defined in eqn (10) of the manuscript. Minima are marked as circles. Results for $\lambda = 0$ are the same as those shown in Fig. 3 of the manuscript.

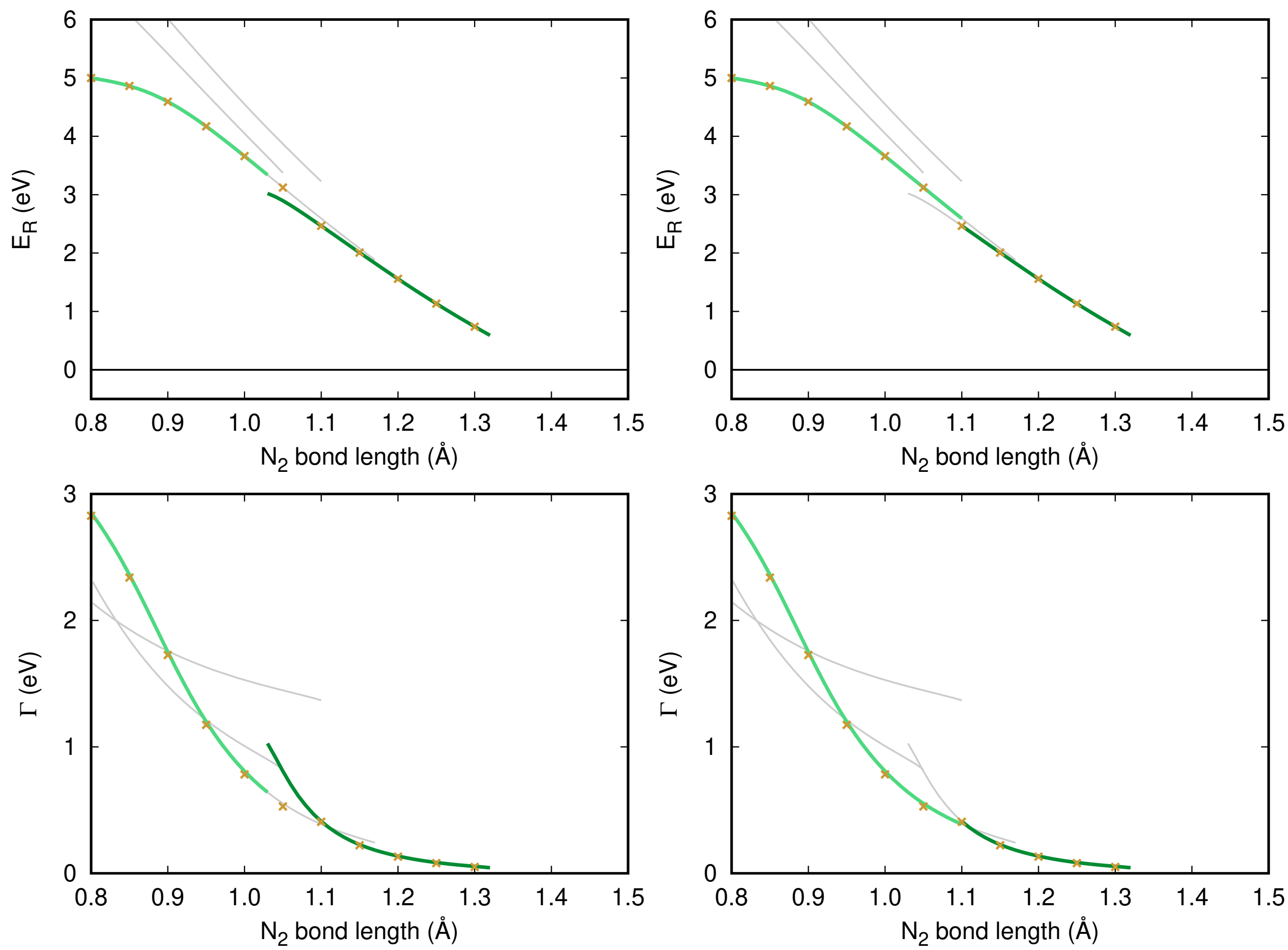

FIG. S9. The resonance position (E_R) and width (Γ) of $N_2{}^-$, calculated with 0th-order CAP/EOM-EA-CCSD/aug-cc-pVTZ+3s3p3d(C), display multiple CAP solutions (gray) that appear and disappear as the bond length varies. The colored curves denote the single solution with the smallest $\eta_{\rm opt}$ (left) or with the smallest velocity (right). Results from Ref. 1 are marked as crosses.

TABLE S8. Resonance position (E_R), resonance width (Γ), optimized CAP strength ($\eta_{\rm opt}$), and its associated velocity [$v(\eta_{\rm opt})$], computed at three bond lengths of N_2^-, for CAP onsets specific to each geometry (flexible), or kept at the equilibrium geometry values (fixed). The onsets are given in Table S9 and were obtained as described in Ref. 1.

Bond length (Å)	Solution	Onset	E_R (eV)	Γ (eV)	$\eta_{\rm opt}$ (10^{-5} a.u.)	$v(\eta_{\rm opt})$ (10^{-3} a.u.)
0.80	*b*	fixed	4.999	2.852	1061	2.16
		flexible	5.094	2.996	884	0.99
	c	fixed	6.718	2.327	5976	17.00
		flexible	6.696	2.443	4510	16.32
	d	fixed	7.527	2.146	27060	14.15
		flexible	7.595	2.496	19800	18.87
1.15	*a*	fixed	2.007	0.227	138	0.35
		flexible	2.007	0.227	142	0.33
	b	fixed	2.081	0.279	615	2.25
		flexible	2.081	0.276	637	2.22
	c	fixed[a]	-	-	-	-
		flexible	2.665	1.316	18300	14.42
1.30	*a*	fixed	0.740	0.055	101	0.53
		flexible	0.740	0.054	112	0.52

[a] The analogous minima falls short of appearing.

TABLE S9. CAP onsets (in a_0) for N_2, determined at the neutral equilibrium geometry taken from Ref. 3, of around 1.098 Å, and at internuclear distances shown in Table S8.

Bond length (Å)	x_0	y_0	z_0
0.80	2.612	2.612	4.063
1.098	2.760	2.760	4.880
1.15	2.787	2.787	5.030
1.30	2.839	2.839	5.465

TABLE S10. Equilibrium distance (R_0) and harmonic vibrational frequency (ω) of N_2^-, and transition energy between the vibrational ground states of N_2 and N_2^- (E_{00}), according to the presently obtained 0th-order (*a*) and 1st-oder (*a*′ and *b*′) solutions of CAP/EOM-EA-CCSD/aug-cc-pVTZ+3s3p3d(C) and to previous calculations.

Method	R_0 (Å)	ω (cm^{-1})	E_{00} (eV)
CAP/EOM-EA-CCSD/aug-cc-pVTZ+3s3p3d(C) (*a*)	1.170	1891	2.178
CAP/EOM-EA-CCSD/aug-cc-pVTZ+3s3p3d(C) (*a*′)	1.166	1844	2.146
CAP/EOM-EA-CCSD/aug-cc-pVTZ+3s3p3d(C) (*b*′)	1.174	1937	2.220
CAP/EOM-EA-CCSD/cc-pVTZ+2s5p2d(A)[a]	1.180	1751	2.211
CAP/MRCISD+Q_P/cc-pVTZ+2s5p2d(A)[a]	1.191	1959	2.154
CAP/XMS-CASPT2/cc-pVTZ+2s5p2d(A)+1d(C)[a]	1.184	1800	2.115

[a] Ref. 15.

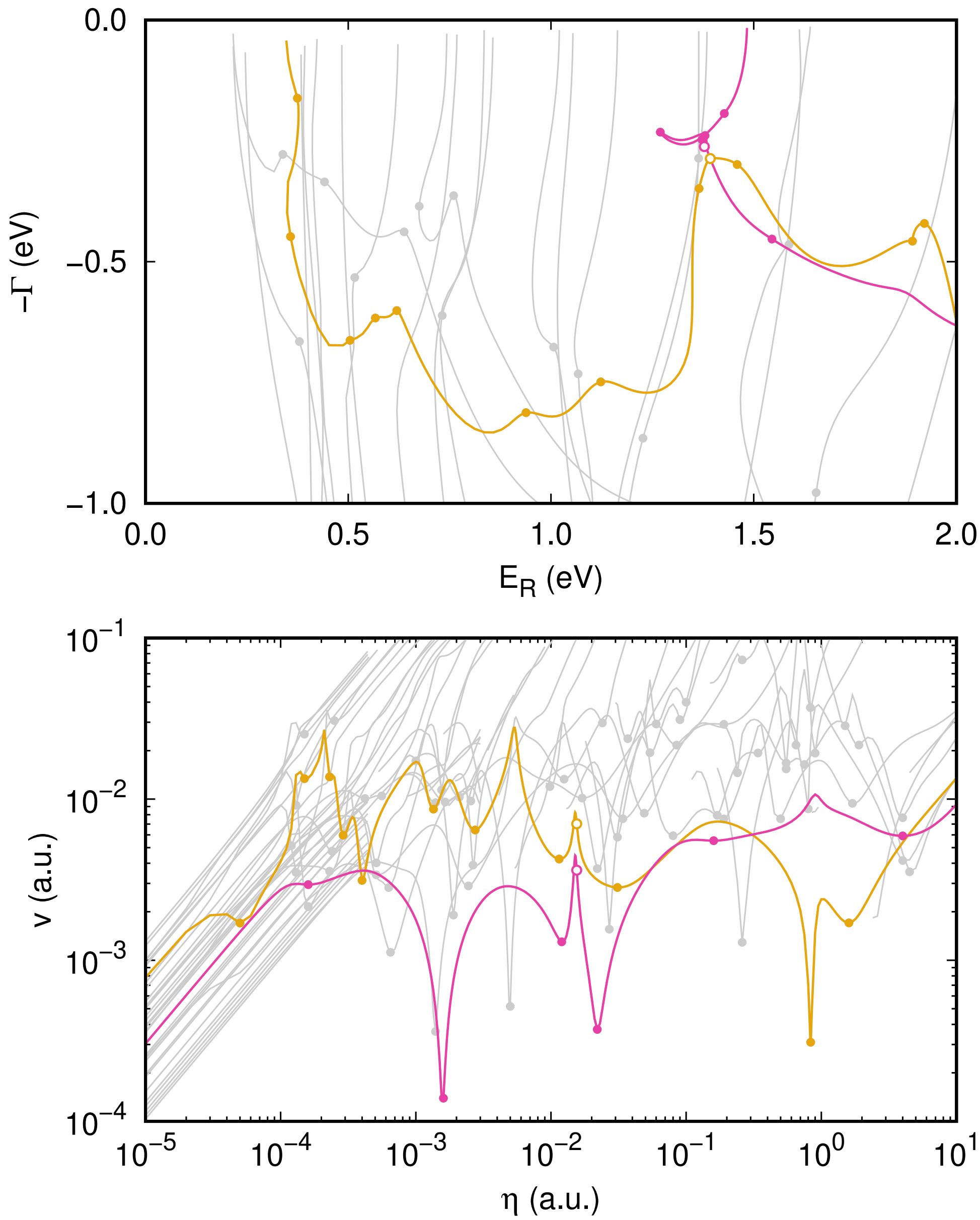

FIG. S10. Zoom-out of Fig. 13 of the manuscript, showing the η-trajectories (top) and velocities (bottom) for the chloroethylene anion at the MEEP geometry of Ref. 4. Those associated with the MEEP quasi-degeneracy are shown in color. The MEEP solutions are marked as open circles. Minima are marked as filled circles.

TABLE S11. Resonance position (E_R), resonance width (Γ), CAP strength (η_{opt}), and its associated velocity [$v(\eta_{\text{opt}})$], for the MEEP solutions and closest CAP solutions of the chloroethylene anion, at the MEEP geometry reported in Ref. 4, calculated with 0th-order CAP/EOM-EA-CCSD/aug-cc-pVDZ+3p(A). The associated η-trajectories and velocities are shown in Fig. 13 of the manuscript.

Solution	E_R (eV)	Γ (eV)	η_{opt} (10^{-5} a.u.)	$v(\eta_{\text{opt}})$ (10^{-3} a.u.)
b_1	1.270	0.232	160	0.14
c_1	1.380	0.239	1200	1.31
MEEP_1	1.378	0.262	1550	3.62
d_1	1.373	0.248	2200	0.37
h_2	1.365	0.349	1150	4.25
MEEP_2	1.393	0.287	1550	7.02
i_2	1.459	0.299	3100	2.83

REFERENCES

[1]T.-C. Jagau and A. I. Krylov, J. Phys. Chem. Lett. **5**, 3078 (2014).

[2]T.-C. Jagau, K. B. Bravaya, and A. I. Krylov, Annu. Rev. Phys. Chem. **68**, 525 (2017).

[3]D. Zuev, T.-C. Jagau, K. B. Bravaya, E. Epifanovsky, Y. Shao, E. Sundstrom, M. Head-Gordon, and A. I. Krylov, J. Chem. Phys. **141**, 024102 (2014).

[4]Z. Benda and T.-C. Jagau, J. Phys. Chem. Lett. **9**, 6978 (2018).

[5]Z. Benda and T.-C. Jagau, J. Chem. Theory Comput. **14**, 4216 (2018).

[6]S. Feuerbacher, T. Sommerfeld, and L. S. Cederbaum, J. Chem. Phys. **120**, 3201 (2004).

[7]Q. M. Phung, Y. Komori, T. Yanai, T. Sommerfeld, and M. Ehara, J. Chem. Theory Comput. **16**, 2606 (2020).

[8]P. Burrow, A. Modelli, N. Chiu, and K. Jordan, Chem. Phys. Lett. **82**, 270 (1981).

[9]K. Stricklett, S. Chu, and P. Burrow, Chem. Phys. Lett. **131**, 279 (1986).

[10]J. R. Gayvert and K. B. Bravaya, J. Chem. Phys. **156**, 094108 (2022).

[11]M. Thodika and S. Matsika, J. Chem. Theory Comput. **18**, 3377 (2022).

[12]L. Burth, F. Kossoski, and P.-F. Loos, J. Chem. Theory Comput. **21**, 11463 (2025).

[13]Y. Damour, A. Scemama, F. Kossoski, and P.-F. Loos, J. Phys. Chem. Lett. **15**, 8296 (2024).

[14]M. Berman, H. Estrada, L. S. Cederbaum, and W. Domcke, Phys. Rev. A **28**, 1363 (1983).

[15]S. Mondal and K. B. Bravaya, J. Chem. Phys. **161**, 024106 (2024).
